# Multicancer screening test based on the detection of circulating non haematological proliferating atypical cells

**DOI:** 10.1186/s12943-024-01951-x

**Published:** 2024-02-13

**Authors:** Natalia Malara, Maria Laura Coluccio, Fabiana Grillo, Teresa Ferrazzo, Nastassia C. Garo, Giuseppe Donato, Annamaria Lavecchia, Franco Fulciniti, Anna Sapino, Eliano Cascardi, Antonella Pellegrini, Prassede Foxi, Cesare Furlanello, Giovanni Negri, Guido Fadda, Arrigo Capitanio, Salvatore Pullano, Virginia M. Garo, Francesca Ferrazzo, Alarice Lowe, Angela Torsello, Patrizio Candeloro, Francesco Gentile

**Affiliations:** 1 Department of Health Sciences, University Magna Graecia, Catanzaro, IT Italy; 2 Department of Experimental and Clinical Medicine, University Magna Graecia, Catanzaro, IT Italy; 3 Department of Chemistry, University of Leicester, Leicester, UK; 4 Pathology Unit Pugliese Ciaccio Hospital, Catanzaro, IT Italy; 5 Unilabs Pathology, Lugano, CH Switzerland; 6 Candiolo Cancer Institute, FPO-IRCCS, Candiolo (TO), Turin, Italy; 7 Società Italiana di Citologia (SICi), AO S.Giovanni-Addolorata, President, Roma, IT Italy; 8 Cytodiagnostic Pistoia-Pescia Unit, USL Toscana Centro, Pistoia, IT 51100 Italy; 9 LIGHT Center, Brescia, IT 25123 Italy; 10 Pathology Unit, Central Hospital Bolzano, via Boehler 5, Bolzano, IT 39100 Italy; 11 Human Pathology Department, Gaetano Barresi University, Messina, IT Italy; 12 Linköping University Hospital SE , Linköping University, Linköping, Sweden; 13 Department of Pathology, Stanford University Hospital, Stanford, CA USA; 14 UOC Oncology, AO San Giovanni, Roma, IT Italy

**Keywords:** Cancer prevention, Liquid biopsy, Multicancer diagnosis, Non heamatological proliferating cells, Predictive model, Supervised machine learning, Neural network algorithm

## Abstract

**Background:**

the problem in early diagnosis of sporadic cancer is understanding the individual’s risk to develop disease. In response to this need, global scientific research is focusing on developing predictive models based on non-invasive screening tests. A tentative solution to the problem may be a cancer screening blood-based test able to discover those cell requirements triggering subclinical and clinical onset latency, at the stage when the cell disorder, i.e. atypical epithelial hyperplasia, is still in a subclinical stage of proliferative dysregulation.

**Methods:**

a well-established procedure to identify proliferating circulating tumor cells was deployed to measure the cell proliferation of circulating non-haematological cells which may suggest tumor pathology. Moreover, the data collected were processed by a supervised machine learning model to make the prediction.

**Results:**

the developed test combining circulating non-haematological cell proliferation data and artificial intelligence shows 98.8% of accuracy, 100% sensitivity, and 95% specificity.

**Conclusion:**

this proof of concept study demonstrates that integration of innovative non invasive methods and predictive-models can be decisive in assessing the health status of an individual, and achieve cutting-edge results in cancer prevention and management.

**Supplementary Information:**

The online version contains supplementary material available at 10.1186/s12943-024-01951-x.

To the editor,

## Background

The strategy to ensure a tangible decrease in cancer morbidity and mortality resides in preventive medicine and the improvement of screening programs. Investment in cancer prevention results in both health and economic impacts. These effects become obvious when one compares the cost of cancer to the cost of the last pandemic, the average direct medical cost of a symptomatic COVID-19 patient being $3,045 [1] vs. an estimated average direct cost to the Health System of a patient with breast cancer being $37.968 [2]. The key to preventive strategies in cancer is the assignment of an individual risk level of developing disease. Historically comprised largely of generalized lifestyle-based recommendations [3], cancer prevention and screening are now evolving to incorporate specific relevance to individual patients by the integration of precision medicine with artificial intelligence (AI) technology [4]. The non-invasive analytical approach favours the development and establishment of proactive screening programs for cancer-related risk assessment and improving early diagnosis platforms. Liquid biopsy (LB) is a major player in this transformation. LB is a non-invasive test (often a blood test) initially developed and employed in the management of cancer patients. In the past decade the use for the LB has transitioned from the control of morbidity in established tumours (tertiary prevention), [5] to secondary prevention for early diagnosis [6] and, more recently, to assess the cancer risk [7]. Research in the liquid biopsy field has highlighted blood as a source of molecular and cellular markers that originate in tissue. Indeed, protocols and devices have been developed to isolate and analyze cell-free DNA [8], circulating epithelial cells [9], endothelial cells [10], and cells with an epithelial and mesenchymal phenotype [11]. All of these biomarkers have proven to be useful in improving the management of cancer patients and more recently have been implicated as a possible marker of tissue damage, the identification of which can be subsequently leveraged, in the cancer screening and prevention context, to introduce and calibrate control measures to limit hypothetical broader risk [12].

Parallel to the development of LB applications in preventive medicine, AI was used to identify an increasing degree of health problems in various segments of the population for a novel cycle of screening programs, including the targeted screening and stratified screening [3, 13].

In this current study, we have hypothesized to use of blood as a source of non-haematological cells which may exhibit signs of atypia and a dysregulated proliferation profile, which together suggest tumour pathology.

## Results

### Comprehensive assessment of cell type-specific differential CD45 expression in blood-derived samples

To assess cell proliferation, cell suspensions derived from blood, after a gradient passage to reduce the blood cell contamination, were cultured for a brief period (short-time culture) revealing non-haematological elements that are typically rare. This approach highlighted proliferation dysfunction, if present, and allowed proliferating cells to be further analyzed, allowing for the evaluation of their degree of atypia and the identification of their tissue of origin [14].

**Fig. 1:**
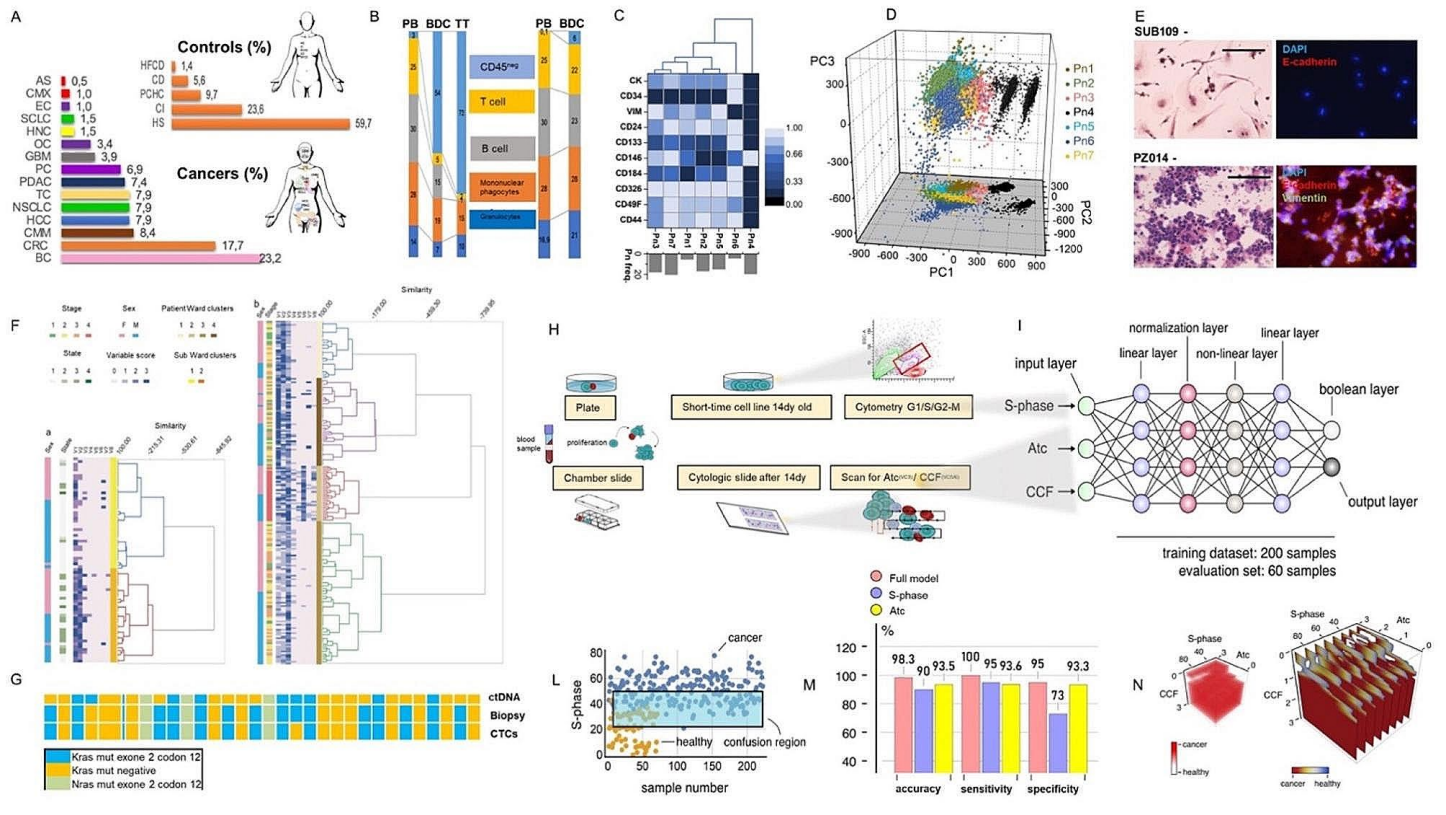
Non-haematological cell features are input/output models to simulate multicancer screening. **A**) Baseline percentage related to the clinical presentation of healthy subjects enrolled as controls (CS) and the type of tumours in the cancer patient group (CP). **B**) Different proportions of CD45 pos/ neg cells in liquid and solid matrices. In **C**) Identification of six patterns (Pn) by heatmap depicting expression levels of each marker on individual cell. Principal Analysis Component (PCA) maps allowed for a multidimensional separation of cell populations in CS and CP groups **E)** Cytological images of blood-derived specimens in CS (SUB109) and CP (PZ014) and immunofluorescence analysis of epithelium-specific cadherin (red) and biomarkers of EMT as vimentin (green) expression **F)** Ward’s clustering cytopathologic features in CS and CP. **G)** mutational profile in order of Kras mutation comparing liquid and tumour biopsy and CTCs with ct-DNA in same colon cancer patients. **H**) workflow starts from blood collection through short time culture to ML application. **I**) Neural model incorporates a linear, non-linear, normalization layer linked together. **L)** Decision boundary or confusion region between the two groups in order of S-phase input **M**) Performance of the neural network model **N**) 3D representation of the model output as a function of cancer features as S-phase, atypical cells (Atc) and cells organized in clusters (CCF)

We have performed blood-derived short-time cultures (BDCs) from control subjects (CS) and cancer patients (CP) (Fig. 1A) (Supplementary file 1: Table S1), enrolled following the project acceptance criteria approved by local government ethical standards with number ID 2013/34, detailed in Supplementary file 2 (Fig. S1-7). Further evaluation of the cell type yielded by the cell cultures was performed by evaluating for the expression pattern of the leukocyte common antigen CD45. This assessment endorsed for the determination of non-haematological and blood cell proportions at the end of short time cultures.

The percentage of non-haematological cells obtained from the CP-BDCs (54% ± 9) was significantly higher compared to CS-BDCs (6% ± 0.9) (*p* < 0.001) (Fig. 1B).

### Clinical implications of intra and inter blood-derived sample heterogeneity

Cultured non-haematological cells were further analyzed with a panel of epithelial and mesenchymal markers: panCK, CD34, Vimentin, CD24, CD133, CD184, CD326, CD49F, CD44, CD146. Levels of antigen cell-surface expression were quantified as mean fluorescence intensity normalized to 1 (Supplementary file 3: Table 2). The heat map in Fig. 1C illustrates the differential expression of marker levels and highlights the seven phenotypic patterns (Pn) that were identified. Each Pn is given by a combination of phenotypic profile displayed by cultured cells. CS showed a sole pattern, Pn4, characterized by negative or low expression of CK, CD34, Vimentin, CD24, CD133, CD184, CD326, CD49F, CD44 and by high positivity for CD146. Contrary, in CP’ BDCs six patterns were identified (Pn1,2,3,5,6,7) by the exclusion of phenotypic redundancy data Fig. 1C. Moreover, cell phenotype in Pn4 showed two levels of CD146 expression Fig. 1D. The presence of reactive endothelial cells positive for CD146 in CS was related to clinical inflammatory conditions detailed in table S1 and previosly observed [10]. According to the clinical impact of heterogeneity in marker expression, the principal CP’s Pns affecting disease-free survival (DFS) and overall survival (OS), were the Pn6 and Pn7, Fig. 1D, with both showing a significant fraction of cultured cells characterized by an epithelial-mesenchymal transition phenotype, Fig. 1E. Pn6 showed a disease free survival (DFS) of 2 months (95% CI, 4–6) and overall survival (OS) of 8 months (95% CI, 6–9) while Pn7 showed a DFS of 5 months (95% CI, 4–6) and an OS of 10 months (95% CI, 8–13) (Supplementary file 4).

### Processing and reporting of cytology specimens from blood-derived samples

Anticipating that the BDCs obtained from CS and CP groups would be distinguishable according to cell tumour heterogeneity, all experimental steps were conducted on chamber slides to obtain corresponding microscope slides. The microscope slides collected were explored and classified in order of cytopathological variables (detailed in Supplementary files 6 and 7). A total score from each specimen was calculated by precise cellularity guidelines (score 1: 1–5 target cells/100 cells; score 2: 5–10 target cells/100; score 3: >10 target cells/100 cells) applied on pathological variables considering the rate of lympho-monocytes (Vc1), endothelial cells (Vc2), atypical cells (Vc3), mitotic figures (Vc4), homotypic (Vc5) and heterotypic cell clusters (Vc6), monocyte macrophages (Vc7) and multinucleated cells (Vc8).

Ward’s hierarchical clustering score-based cytopathological variables method (Fig. 1F) revealed a high level of segregation between CS (Fig. 1F-a) and CP specimens (Fig. 1F-b) and a high level of correlation with tumour progression (Spearman coefficient rho = 0.5).

### Clinical impact of blood-derived samples proliferation profile

At the same time, the analysis of the proliferation profile of cultured cells from the two groups showed a significant difference expressed as a percentage of cells in S-phase (*p* = 0.001). In CP ‘ BDCs, the percentage of cells in S-phase correlated with cancer stage (*p* = 0.0004) and OS (*p* = 0.001) (Supplementary file 4) (Fig. 1H). Further experiments on mutational pattern of the experimental cells isolated from cancer patients, showed a linear correlation with the mutational signature of autologous samples from the primary tumour biopsy (k = 0.9) and ct-DNA (k = 0.5) Fig. 1G (Supplementary files 8 and 9).

### Supervised machine learning (ML) model based to decode cancer features

Finally, we used a supervised machine learning (ML) model based on artificial neural networks to decode cancer features and improve cancer diagnosis.

ML was used to examine whether all or some of cancer cell signatures identified in this study could offer guidance to the underlying diagnosis.

The neural networks model was composed by a sequence of several layers, as described in the Supplementary File 5. Variables passed to the model as input data were: (i) the value of S-Phase, (ii) the grade of atypia – corresponding to cyto-pathological variable (Vc) Vc3, (iii) and the grade of cluster formation (CCF), identified by the variables Vc5, Vc6 and expressed as a percentage or a positive integer Vc3, Vc5, Vc6 (Fig. 1I).

The model’s output was a Boolean variable [15] with two possible values indicating whether the patient has developed aggressive forms of cancer (1) or not (0) (Fig. 1I). The performance of the model was measured using three different metrics, i.e. accuracy, sensitivity, and specificity, defined as (i) the proportion of the correct predictions among the total number of examined cases, (ii) the proportion of positive results that were true positives and (iii) the proportion of negative results being true negatives (Fig. 1L). The principal ML outputs identified were atypia (Vc3) and proliferation rate (S-phase), which, when analyzed together, were able to allow a cancer detection resulting in 98.8% accuracy, 100% sensitivity, and 95% specificity (Fig. 1M) with high grade of resolution (Fig. 1N).

## Conclusion

In summary, this research suggests the existence of a fundamental logic underlying the complexity of early blood-based diagnosis. The logic is based on the presence of specific populations of non-haematological cells that can be characterized in terms of atypia, proliferation, and tissue origin, as previously demonstrated [14]. As research advances, this effective assessment methodology is expected to demonstrate its unique advantages, such as specificity and repeatability, in clinical translation.

Considering that the turnaround time (TAT) and cost for a single test are both equivalent to the TAT and economic impact of a conventional cytological examination, doctors may apply this approach not only to monitor patient’s response to therapy (in line with the traditional vocation of LB), but also to formulate personalized surveillance and general prevention, if applicable, based upon individual patient characteristics/risk factors.

This test represents a real collaboration between LB and AI that has resulted in a strategic cancer prevention roadmap. This roadmap is able to identify individual cell features/phenotypic patterns which can highlight the subpopulation of patients at higher risk of developing cancer. We propose that those identified through this stratified screening program have an opportunity that allows for early diagnosis - the offensive front line in fighting the war on cancer- and subsequent treatment which, if effective, are anticipated to result in a rapid return to baseline values.

### Electronic supplementary material

Below is the link to the electronic supplementary material.

Supplementary Material 1

Supplementary Material 2

Supplementary Material 3

Supplementary Material 4

Supplementary Material 5

Supplementary Material 6

Supplementary Material 7

Supplementary Material 8

Supplementary Material 9

## Data Availability

All data underlying the findings reported in this study and three categories of image data of cell specimens (obtained by applying Charactex protocol on blood sample taken from breast, colon and NSCLC patients) are deposited in the public data repository OSF under the name “Multicancer screening test ” (10.17605/OSF.IO/4356F).

## Abbreviations

CRC: Colorectal cancer
GBM: Glioblastoma
HNC: Head and neck carcinomas
HCC: Hepatocellular carcinoma
PDAC: Pancreatic ductal adenocarcinoma
NSCLC: Non-small-cell-lung-cancer
SCLC: Small cell lung cancer
BC: Breast cancer
CMM: Cutaneous malignant melanoma
EC: Endometrial cancer
OC: Ovarian cancer
PC: Prostate cancer
TC: Thyroid cancer
CM: Cardiac myxoma
AS: Angiosarcoma
HS: Healthy subjects
CI: Chronic inflammation
PCHC: Previous clinical history of carcinoma
CD: Cardiovascular disease
HFCD: Hereditary/familiar cancer disease
BDCs: Blood-derived cultures
TTB: Tumor tissue biopsy
PB: Peripheral blood
ct-DNA: Circulating tumor DNA
CTCs: Circulating tumor cells
ATC: Atypical Cell or Vc3
CCF: Cell cluster formation or Vc5/6
OS: Overall survival
DFS: Disease free survival
TAT: Turnaround Time
