# Supplementary material for: Multicancer screening test based on the detection of circulating non haematological proliferating atypical cells
Source: Mol Cancer. 2024 Feb 13;23:32. doi: 10.1186/s12943-024-01951-x (PMC10863189; doi:10.1186/s12943-024-01951-x)
Supplement: Supplementary file 3 — Supplementary Material 3 [file 12943_2024_1951_MOESM3_ESM.pdf]

Additional file 4 : Supplementary file.pdf. Clinical impact of blood-derived cell signatures and proliferation profiles

Correspondence to: nataliamalara@unicz.it.

This PDF file includes:

Supplementary Text
Figs. S8 to S10

Charactex clinical workflow

276 Peripheral blood samples were collected between December 18, 2013, and October 18, 2018, by the CHARACTEX project, the number of identification 2013.34 of which 205 were cancer patients of which 14 arrived without a cancer diagnosis, 191 with a previous cancer diagnosis, and 72 were healthy subjects.
Of the 14 cases without cancer diagnosis through the application of the CHARACTEX protocol were identified 5 new cancer cases (1 thyroid undifferentiated cancer, 2 melanomas, 1 pancreas adenocarcinomas, and 1 NSCLC) and 3 cases positive for CTCs that developed cancer within 1 year (1 colon adenocarcinoma, 1 intra-ductal breast cancer, 1 mucinous ovary cancer). These 8 new cases of cancer were positive for the presence of
CTCs at diff-quick, immunocytochemical assay and were characterized by S phase>30%. All cases were confirmed by successive histopathological examinations on primary tumour lesions. Moreover, were identified 6 cases positive for CTCs characterized by lower S phase (<30%) and negative for clinically evident cancer lesions. 4 cases of the 6 become negative for CTCs within 1 year. The remaining 2 cases were repeated every 6 months for blood sampling and clinical control. Their S-phase remains at low values (<30). In all cases with a previous diagnosis of cancer (191), the cytological examination on CTCs confirmed the first diagnosis performed on tumour tissue. Submitted to anticancer treatments 32 patients repeated blood sampling, all after surgical tumour removal and 14 after chemotherapy. Finally, the 72 healthy voluntaries confirmed the negativity

for cancer diagnosis and their blood-derived cell cultivation was characterized by the absence of sporadic presence of atypical cells (0,5 cell/1000 cells) and proliferation profile (<30%)

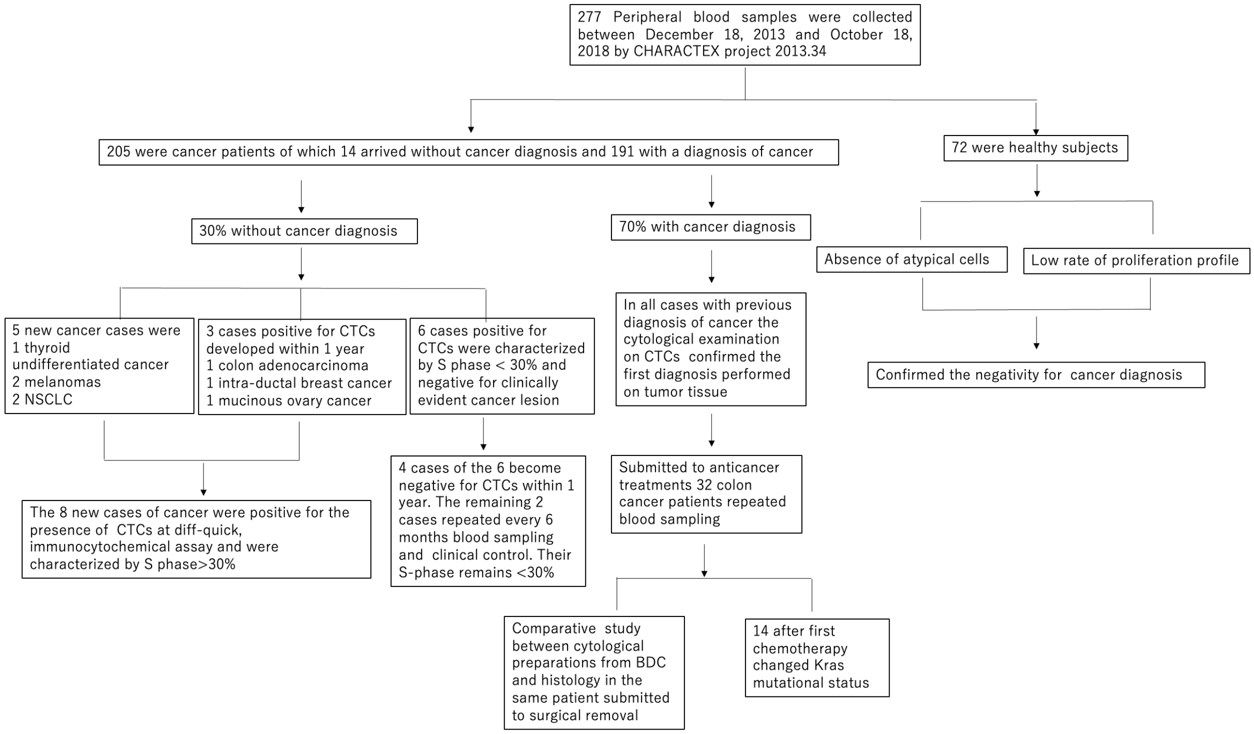

Figure S8: CHARACTEX clinical workflow. The protocol was performed on 277 Peripheral blood samples collected between December 18, 2014 and December 18, 2018

Disease-free survival (DFS) for cancer cohort to phenotypic patterns (Pn)

The corresponding median of the disease-free survival (DFS) for the cancer cohort and relative Pn was performed. DFS for CP with Pn1 was 17 months (95 % CI, 14-22), Pn2 was 10 months s (95 % CI, 6-13), Pn3 was 10 months s (95 % CI, 9-12), Pn5 was 16 months s (95 % CI, 12-20), and for patients with Pn6 was 2 months (95 % CI, 4–6), finally Pn7 was 5 months s (95 % CI, 4-6). We detected a significant prognostic value for the interaction using a univariate Cox proportional hazard regression model (p = 0.003). The median the overall survival for patients within the Pn1 was 22 months (95 % CI, 20-24), for Pn2 was 17 months s (95 % CI, 14-19), for Pn3 was 18 months s (95 % CI, 18-19), for Pn5 was 21 months s (95 % CI, 19-23), and for patients within Pn6 was 8 months (95 % CI, 6–9), finally, for Pn7 it was 10 months s (95 % CI, 8-13). We detected a significant prognostic value for the interaction using a univariate Cox proportional hazard regression model (p=0.008)

Disease-free survival (DFS) for cancer cohort to S-phase fraction (SPF)

In the prognostic term, the median of disease-free survival for patients with an S phase <50% was 12 months (95 % CI, 10-14), with an S phase >50% was 6 months (95 % CI, 4-7) . The median of the overall survival for patients with an S phase <50% was 18 months (95 % CI, 17-20), and 12 months for those with an S phase >50% (95 % CI, 11-14). These data suggest a prognostic role in the determining of overall survival in the cancer cohort of the expanded CTCs-SPF confirmed by significant prognostic value for the interaction using a univariate Cox proportional hazard regression model (p = 0.001).

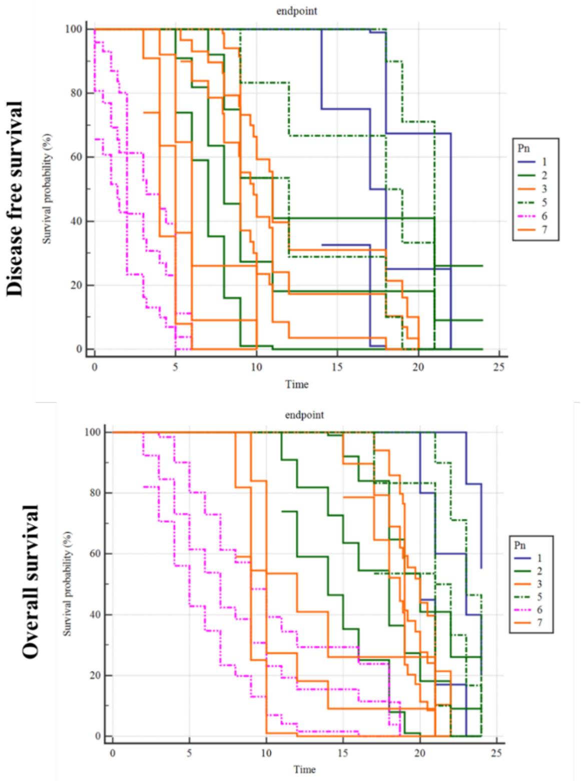

Fig. S9. Disease-free survival and overall survival curves according to CTCs phenotype. Estimated survival times from Kaplan-Meier plot inspection/ Kaplan-Meier curves of disease-free survival and overall survival in the population of cancer patients stratified for phenotypic subtype Pn (Pn1-6).

Disease free survival

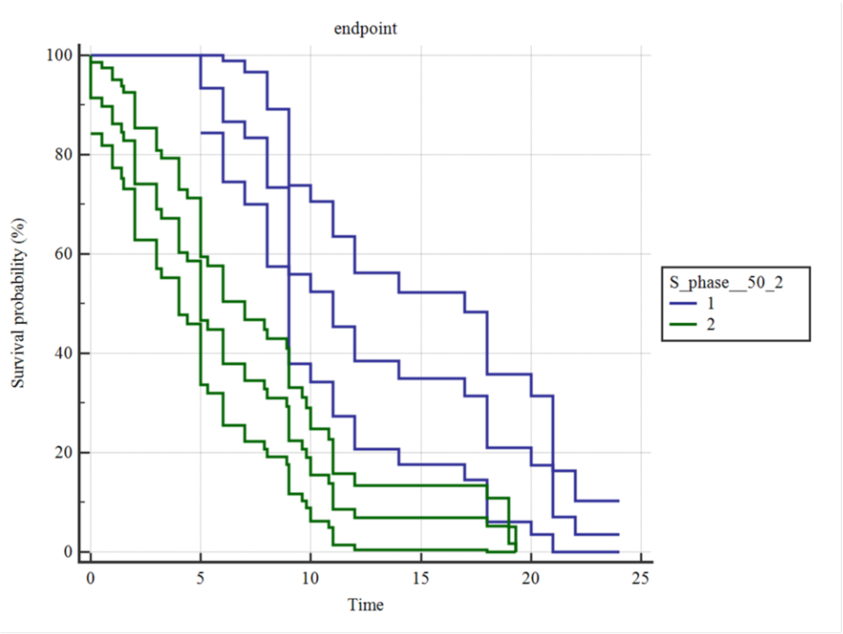

Overall survival

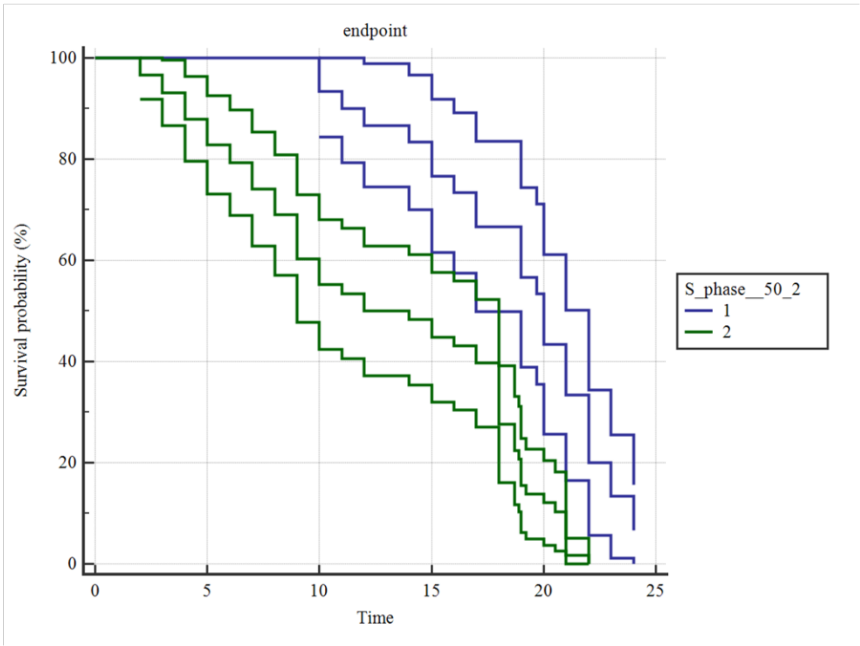

Fig S10. Disease-free survival and overall survival curves according to CTC proliferation. Estimated survival times from Kaplan-Meier plot inspection/ Kaplan-Meier curves of disease-

free survival and overall survival in the population of patients stratified for the S-phase < 50% (1) >50% (2)
