# Supplementary material for: Multicancer screening test based on the detection of circulating non haematological proliferating atypical cells
Source: Mol Cancer. 2024 Feb 13;23:32. doi: 10.1186/s12943-024-01951-x (PMC10863189; doi:10.1186/s12943-024-01951-x)
Supplement: Supplementary file 4 — Supplementary Material 4 [file 12943_2024_1951_MOESM4_ESM.pdf]

Additional file 2 : Supplementary file.pdf. Project information and validation.

Correspondence to: nataliamalara@unicz.it.

This PDF file includes:

Supplementary Text
Figs. S1 to S7
Tables a-c

Charactex (characterization of circulating tumour cells and expansion) methodology

The potential of CTCs, as minimally invasive biomarkers for predicting patient prognosis and monitoring relapse and as targets for precision therapies, has been demonstrated in several studies but their practical application is limited by their rarity and by their limited cell expansion rates and cell yields. Standardization of a unique methodology for in vitro expanding cancer cells isolated from blood is essential for cancer studies. Several studies have shown that it is possible to obtain CTC cell lines from cancer patients and use them i. to diagnose intracranial tumours not otherwise reachable because located in sensitive areas of the brain [5], ii. to accomplish drug discovery for personalized treatment or to monitor the response to anticancer drugs in vitro iii. for examining whether drug sensitivity is consistent with the patient's clinical history. The proposed Charactex (methodology, used in our laboratory since 2013, is efficient in obtaining primary short-time CTC lines: the resulting expanded cell populations describe the heterogeneous characteristics of the original neoplasm about their functional, genotype and phenotype traits.

Primary cell culture is the ex vivo culture of live cells isolated from a multicellular organism. Primary cell cultures, unlike secondary cell cultures, are considered more representative of tissues in vivo. These cultures have several advantages over general cell lines, such as a better representation of the cellular heterogeneity of the tissues, a more faithful transcriptomic profile and proteomics (especially when grown in 3D) and more realistic functionality, including responses to drugs. The heterogeneous composition of primary cultures has in itself a pitfall which is the homogeneous constitution of the tissue from which it comes or which is to be reproduced in vitro. Unlike immortalized cell lines, they become homogeneous through the natural selection of specific subpopulations, undergo a genetic derivation and acquire genetic aberrations. On the other hand, the primary culture cells abandon an adequate reproductive and nutritional cycle, and, after a certain number of divisions, they acquire a senescent phenotype, leading to apoptotic death. During this lifespan, the in vitro conditions should favour the prosperity of the cellular component of the original tissue, while respecting its heterogeneous composition, to assess if it can be considered consistent with its tissue source. In this way, “contamination” by different cell types than those of interest, which might be favoured by the growth conditions, should be limited, keeping to a minimum the misidentification ratio.

Misidentification of cultured human cells within a primary culture, in general,

represents a problem dependent on many variables including the duration of in vitro permanence. According to the Guidelines for Research—Notice Regarding Authentication of Cultured Cell Lines (National Institutes of Health 2007), it is necessary to establish an initial profile on the earliest material; subsequently, the consequent treatment standards should follow the signature of an informed consent obtained following the procedures for the universal voluntary acceptance. In turn, this acceptance will foster the reproducibility and comparability of the research protocol. To assess the primary short-time culture it is therefore vital that adequate records are kept – starting from the time of isolation of the tissue, by detailing the origin, characteristics, and handling procedures of the cell line. If a cell line becomes incorporated into a procedure that requires validation of its components, then its authentication becomes crucial.

Characterization of a cell line is crucial for determining its functions as well as proving authenticity. In blood-derived cultures, special attention must be paid to the possibility that the cell line has become cross-contaminated with residual haematological cells. An actively growing cancer cell line can outgrow another haematological cell component characterized by a slower proliferative rate due to its untransformed condition. On the contrary, in the presence of actively proliferating haematological cells, attempts to correlate cell behaviour with the tissue or tumour of origin might be invalidated by cross-contamination if not accompanied by cytomorphological immunocytochemical and/or other ancillary tests (e.g.: cytofluorimetry, immunofluorescence, FISH, etc).

The presence of haematological contamination in the Charactex protocol is evaluated by analyzing the expression of the CD45 pan-leukocyte antigen. The presence of a positive CD45 proliferating cell population in the blood-derived culture is an indirect indication of the degree of contamination of the culture and, therefore, of the degree of reliability of the molecular analysis. A CD45 negative population greater than 60% indicates a low degree of haematological contamination. With this premise, short-term cultures with a negative CD45 cell density> 60% were analyzed to carry out the subsequent authentication procedures. The specific technique/s used for characterization depends on the type of work being carried out; lineage markers help establish the relationship of a particular cell line to its tissue of origin, and DNA profiling, or analysis of gene expression, is likely to be most useful. Cytology Laboratories may prefer to use chromosome analysis coupled with FISH or the single nucleotide polymorphism array (SNPa) method, also referred to as molecular karyotyping. The SNPa method is a sensitive technology used to perform high-resolution genome-wide DNA copy number analysis and to detect segmental regions of homozygosity, known as regions of copy-neutral loss of heterozygosity (CN-LOH) (figure S1).

Based on these considerations, the identification of the short-term expanded lines was based on the analysis of the cells at the end of the 14 days of culture. Evaluations performed on the 7th day of culture showed cellular changes which already differed from those present in the seeded cells at time zero and that these changes remained qualitatively stable if the in vitro culture time is limited at 14 days and no longer. The culture duration guarantees that the cell population is gently stimulated to expand in vitro with the minimum possible impact on the adaptive diversification of the cells, as shown below. Authentication analysis was performed on cell samples cultured at the end of 14 days, with a haematological component <20%, isolated from the peripheral blood of cancer patients for whom tissue biopsy material from the primary lesion was also available. The SNPs analysis on DNA extracted from cultured CTCs of selected

cases of thyroid carcinoma revealed LOH (segment length 1500000, marker count 500) (Figure S2).

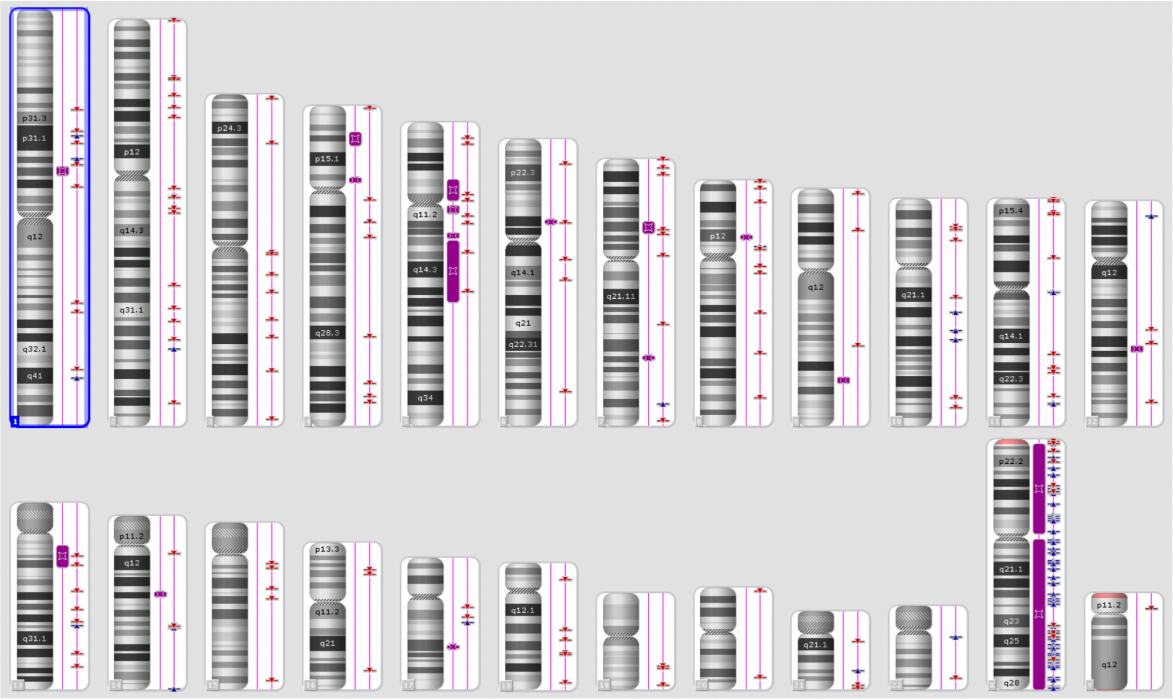

Figure S1: Karioview. Karioview of blood-derived cultured cells from a patient who underwent thyroid resection. Setting Type: smoothing on, joining 50 markers, and 200kb.

Moreover, to investigate transcriptomic changes in the shortly expanded CTC-cell lines compared to their corresponding tissue biopsy, RNA sequencing analysis was performed, including identification and characterization of differentially expressed genes (DEGs) between the two genetic lines. In particular, the cases selected here were: (i) glioblastoma, and (ii) thyroid cancer. Public web server tools for high-throughput genomics data analyses were employed to dissect the identified DEGs for functional interpretations and to select candidate DEGs functionally relevant to provide some insights on the potential different mechanisms of chemo-resistance in two different populations of cancer cells in the same patient.

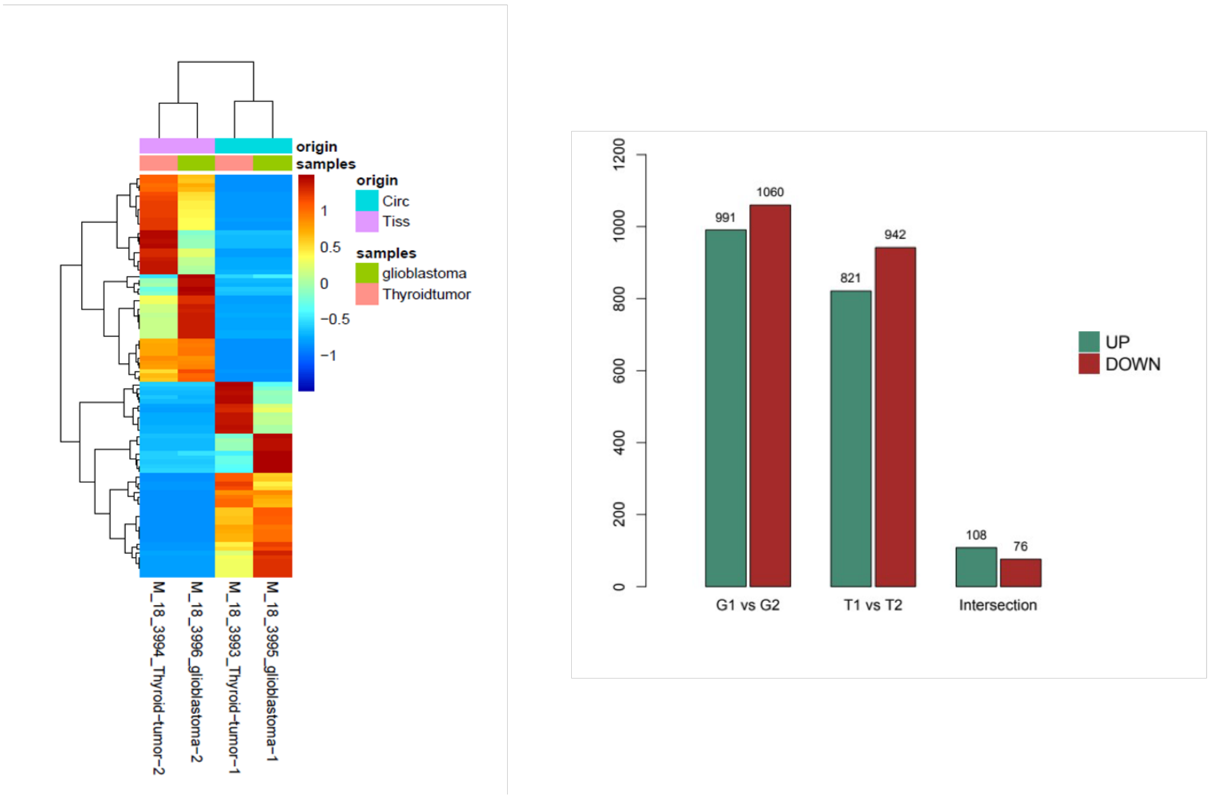

figure S2: DEG overview G1 vs G2 and T1 vs T2 interception: To investigate transcriptomic changes in the CTC-cell lines with respect to relative Tissue biopsy, RNA sequencing analysis was performed, including identification and characterization of differentially expressed genes (DEGs) between the two genetic lines: short-time CTC-cell lines (Circ) versus tissue-derived tumour cells (Tiss). Public web server tools for high-throughput genomics data analyses were employed to dissect the identified DEGs for functional interpretations and to select candidate DEGs functionally relevant to provide some insights on the potential different mechanisms of chemo-resistance in two different populations of cancer cells in the same patient.

Validation of step-by step Charactex protocol (figure S3)

- **Sampling.** To assess the pre-analytical phase in terms of potential external interfering factors with the procedure a series of simulations were performed using stable Green Fluorescent Protein (GFP)-expressing cancer cell lines detectable by cytometry. The GFP-expressing cancer cells were obtained using the methods reported in reference [3], added to the peripheral blood of donors without cancer disease but with different values of Haematocrit (Hct), recovered in the gradient phase as previously described [3-6], and analyzed. The analytical coefficient of variation (CVA) was calculated for each GFP-expressing cancer cell line added to blood samples with, respectively, a higher and lower value of Hct to the physiologic range indicated for adult males (38-52%) and females (36-46%) individuals. A previous study [3] had shown the physiological variability of non-H Haematological (NHC) yield in terms of intra-subject biological coefficient of variation related to the sex and age of the donors (CVI). Moreover, the

calculation of CVA and CVI allows the evaluation of the imprecision of the assay. Formulae for the calculation of the reference CVA (imprecision thresholds) have been applied as already reported and allowed the definition of the assay performance as "optimal" (CVA≤11.6), "desirable" (CVA≤23.3), and "minimum" (CVA≤34.7).

In the case of the method here presented, CVA values (15.7 % for females and 14.6 % for males) resulted very close to the respective optimal reference value (≤ 11.6). In the case of cancer patients undergoing anticancer treatment, the timing to perform the phlebotomy influences the measured CVI in the function of the viability of cellular samples. In the current experiment, a CVI value of 13% was obtained (95% interval 7,3 to 19) for a culture span of 3 days and a CVI value of 17% was obtained (95% interval 9,3 to 25) after a culture time started 18 days after the last anticancer treatment (chemo, chemo/radio, surgery). Conversely, the CVI obtained respectively at 3 and 18 days of culture was 59% (95% interval 43,3 to 77) and 16% (95% interval 12,3 to 20). On these bases, the individual minimal residual disease should be assessed >96h since the last administered anti-neoplastic treatment.

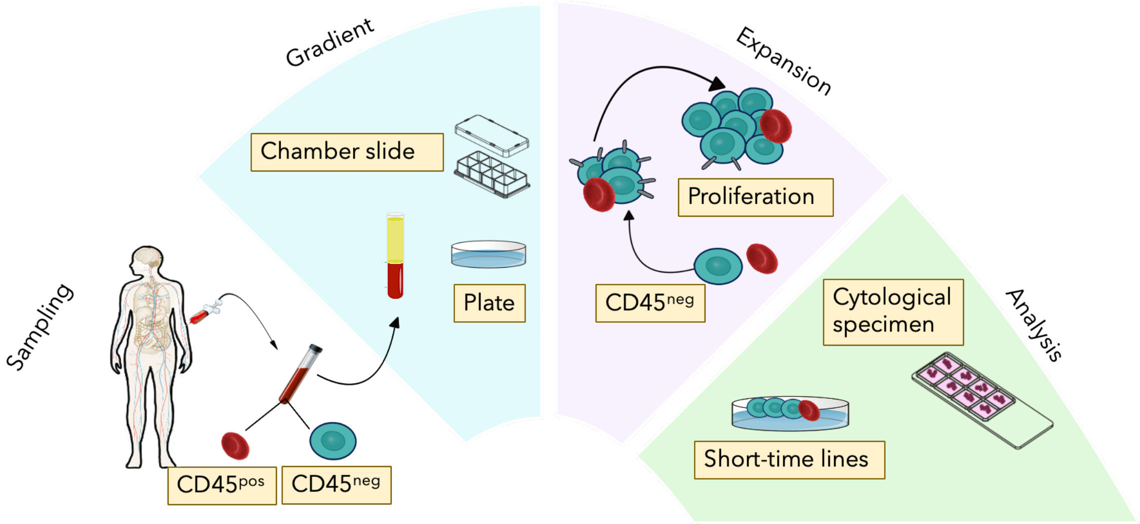

Figure S3: **Charactex's Flow-chart**

- **Gradient phase.** The protocol has been investigated for resolution, sensitivity, specificity, and purity. To test resolution, simulation experiments were performed using detectable tumour cells derived from secondary lines. Each cancer cell secondary line was infected with pAdenoVator-CMV-IRES-GFP reporter, as described in previous reports (7, 11, 12, 13). In particular, the following cell lines were used as references: MCF-7 for breast carcinomas; Lncap for prostate cancer; U373-MG for glioblastoma; Skov for ovarian cancer; A549 for adenocarcinoma alveolar basal epithelial cells; HCT-116 for colon cancer; ECv304 for transformed endothelial cell line derived from Japanese human umbilical vein; CAL62 for thyroid carcinoma; PSN1 for pancreatic cancer and A375 for malignant melanoma. The resolution was calculated using the formula:

$$resolution = (GFP\ cancer\ cells\ added\ x\ counted\ \ GFP\ cancer\ cells) \\ /(number\ of\ acquired\ events\ at\ cytometric\ analysis)$$

The minimal concentration of GFP-pos cancer cells recovered in a 5ml volume of a peripheral blood sample from healthy subjects was of 8 x 10^3 cells. The values of resolution (the number of cancer cells / 5 ml of entire blood) were determined as the mean of five repeated experiments for each type of tumour (Table a).

The sensitivity of the method was calculated through the formula:

$$sensitivity = (True\ positive\ Circulating\ Tumour\ cells\ ) / True\ positive(CTCs\ + false\ negative\ CTCs\ ).$$

The true positive CTCs correspond to the percentage of circulating cells presenting the combined negativity for CD45 and positivity for the antigens specifically referencing the type of tumour as shown in Table b. In particular, if panCK expression is considered as a reference of CTCs:

$$sensitivity\ for\ panCKpos\ CTCs = \frac{PanCKposCD45neg\ CTCs\ \ (\%)}{(PanCKposCD45neg\ (\%)CTCs\ ) + \ PanCKnegCD45negCTCs\ (\%))}$$

The specificity of the methodology was calculated through the formula:

$$specificity = (True\ positive\ CTCs)/\ (True\ positive\ CTCs\ + False\ positive\ CTCs\ )$$

and using the expression of PanCK as a reference of the true CTC became:

$$specificity\ for\ panCKpos\ \ CTCs = \frac{PanCKposCD45neg\ CTCs\ \ (\%)}{(PanCKposCD45neg\ (\%)CTCs\ ) + \ PanCKposCD45posCTCs\ (\%))}$$

The purity, or the percentage of pure CTC, was calculated through the formula

$$purity = (Circulating\ tumour\ cells\ true\ positive)/\ (CTCs\ true\ positive + \ CTCs\ false\ negative\ + \ CTCs\ false\ positive)$$

the formula for a positive CTCs population characterized by a positivity (e. g. for pan CK again) became:

$$purity\ for\ panCKpos\ \ CTCs = \frac{PanCKposCD45neg\ CTCs\ \ (\%)}{(PanCKposCD45neg\ (\%)CTCs\ ) + \ PanCKposCD45posCTCs\ (\%) + \ panCKnegCD45neg\ CTCs\ (\%))}$$

The mean value corresponding to the sensitivity of the method. The sensitivity, calculated for all type of tumours, was 90.5%, the specificity 68.75%, and the purity 73.8%.

Secondary cancer cell lines	Resolution (mean)
Mcf-7	7,4
Lncap	3,2
U373	14
Skov	4,4
A549	3,8
HCT116	5,8
ECV304	11
CAL62	8
PSN1	2,6
A375	2,8

Table a: **Resolution value of the working density phase.** Resolution value of the working density phase for type of cancer: simulation experiments with cancer cell lines. To detect the fraction within the gradient procedure to collect for any type of human carcinoma, we performed a series of simulation experiments. We used cancer cells infected with pAdenoVator-CMV-IRES-GFP reporter as previously described (supplementary data in Malara et al 2016). In particular, MCF-7 was used as reference for breast carcinomas, Lncap for prostate cancer, U373 for glioblastoma, Skov for ovarian cancer, A549 for adenocarcinomic alveolar basal epithelial cells, HCT-116 for colon cancer, ECv304 for transformed cell line derived Japanese human umbilical vein, CAL62 for carcinoma thyroid, PSN1 for pancreatic cancer and A375 for malignant melanoma. The resolution of the method applied, consisting in of the retrieval number of GFP-Cancer cells in the working density phase compared to the starting number of added GFP-Cancer cells, was calculated by the formula: resolution =(positive added x positive counted)/(number of acquired events at cytometric analysis); as previously reported in (supplementary data in Malara et al 2016). The minimal concentration of GFP-Cancer cells added in a volume of peripheral blood sample of 5ml of healthy subjects was of 8 x 10^3 cell and then analyzed at cytometer. The resolution values (number of cancer cells / 5 ml of entire blood) are the mean of five repeated experiments.

Type of tumour	Sensitivity (%)	Specificity (%)	Purity (%)
BREAST CARCINOMA	85	58	69
COLON CANCER	91	87	75
NSCLC	90	84	69
PROSTATE CANCER	88	74	83
THYROID CANCER	81	59	71
PANCREAS CANCER	95	54	87
MELANOMA	97	87	67
GLIOBLASTOMA	97	89	70

Table b. Sensitivity, specificity and purity of CTCs for type of human carcinomas.

The sensitivity, specificity and purity of the methodology were calculated through the formula employing mean values (expressed in percentage) for each CTCs subset of antigens expressed identified by the combined negative expression for CD45. The mean value -corresponding to the sensitivity of the method- calculated for all tumours, was 90.5% the specificity was 68.75% and the purity was 73.8 %.

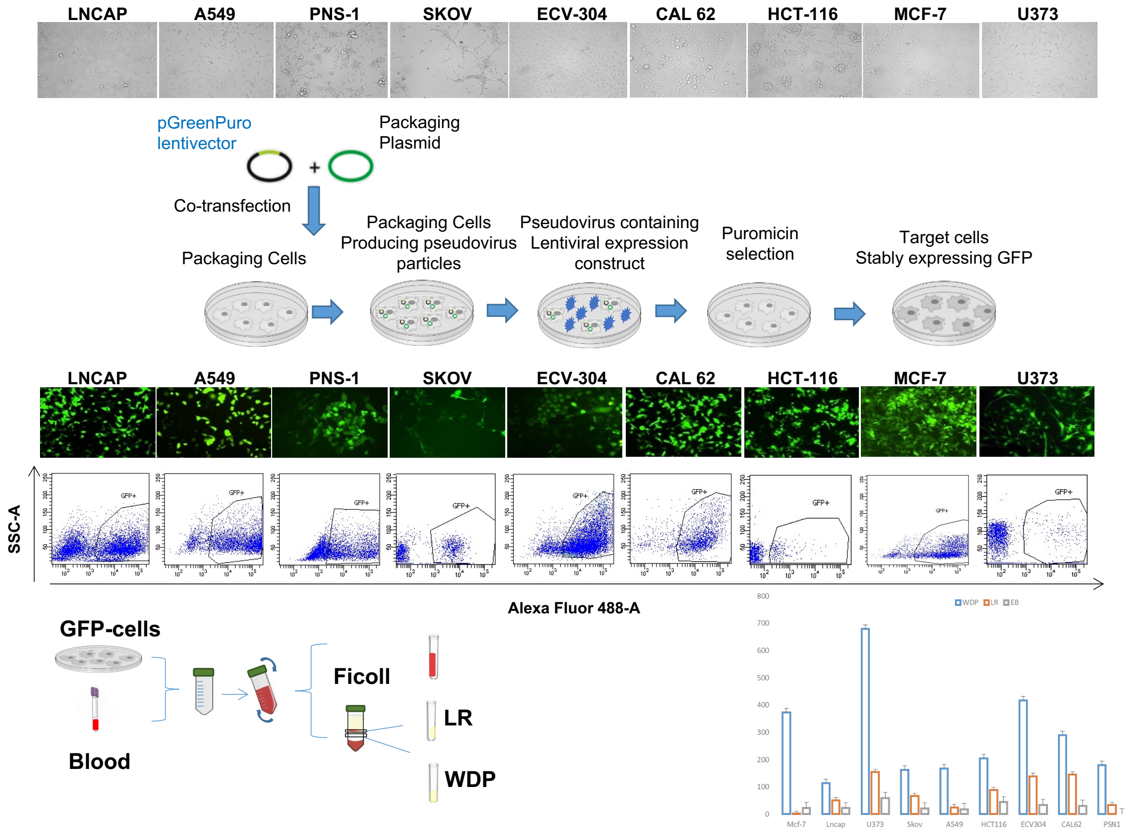

Figure S4: **Assessment of gradient phase.** A list of secondary human GFP-expressing cancer cell lines were used as model to assess the density phase enriched for cancer cells. MCF-7 was used as reference for breast carcinomas; Lncap for prostate cancer; U373-MG for glioblastoma; Skov for ovarian cancer; A549 for adenocarcinoma alveolar basal epithelial cells; HCT-116 for colon cancer; ECv304 for transformed endothelial cell line derived from Japanese human umbilical vein; CAL62 for thyroid carcinoma; PSN1 for pancreatic cancer and A375 for malignant melanoma. The working density phase (WDP) was identified comparing the MFI-Alexa Flour 488-A in each line to the lymphocyte ring (LR). The graph confirm that high MFI-Alexa Flour 488-A is retrieved in WDP (blue) respect to the LR (orange) or whole blood (green) with a significant difference (p<0.005)

Note: GFP; green fluorescent protein; LR, lymphocyte ring; WDP; working density phase

- **Expansion.** The CTCs before the culture expansion are counted using as a reference the haematological cells using the formula:

$$CTC/ml\ peripheral\ blood\ = True\ positive\ \ Circulating\ tumour\ cells\ -\ (CTC\ Isotype\ + false\ negative\ CTCs\ \ + false\ positiveCTCs\ )/Lympho\ Count\ (CD45pos)\ \times absolute\ lymphocytes\ count\ \times\ 1000$$

The number of true positive CTCs corresponds to the CD45 negative cells and (e.g.) PanCK positive (or other specific antigen for type of tumour tissue) minus the number of non-specific events -given by the number of events linking the antibody isotype for PanCK in this example, plus the false negative and positive CTCs. The result is then divided by the value of CD45 positive events counted during the cytometric analysis, multiplied by the number of absolute lymphocytes as from the white blood cell count performed on the day of blood sampling. Often the CTCs count can have a negative value and CTCs become visible only after the brief in vitro expansion. In this case, to calculate the value of CTCs in the blood starting from their value obtained after culture, we use the following formula:

$$CTC/ml\ peripheral\ blood\ = (True\ positive\ CTCs\ \ -\ (CTC\ Isotype\ + False\ negative\ CTCs\ \ + False\ positive\ CTCs\ ))/\ CE/Lymph\ Count\ (CD45pos)\ \times absolute\ lymphocytes\ count\ \times\ 1000$$

Where CE corresponds to the coefficient of expansion specifically adapted to this type of cell culture:

$$Coefficient\ of\ Expansion =$$

$$n^{\circ}\ cells\ attached -\ n^{\circ}\ haematological\ cells\ attached/n^{\circ}\ seeded\ cellsX(Sphase\ value)/100$$

The coefficient of expansion formula takes into account the portion of normal haematological cells surviving in vitro at the end of the culture. Moreover, the number of seeded cells must be considered in proportion to the effective fraction of proliferating cells. The S-phase determination was performed using the BD cell cycle Kit. The evaluation of cell cycle phase distribution is also used to assess the spontaneous apoptosis fraction as the preG0-G1 phase is useful to evaluate the responsivity before in vitro treatment

Table c. Yield of plated human circulating cancer cells at 14 days of culture.

The yield of cancer cells in adhesion and spontaneous sphere formation is reported in percentage as a mean (± S.D.). The number of examined cases for each type of malignancy is reported according to the TNM tumour stage system in localized (T1+T2) and metastatic (T3+T4) (Tumour-Node-Metastasis, UICC-2009).

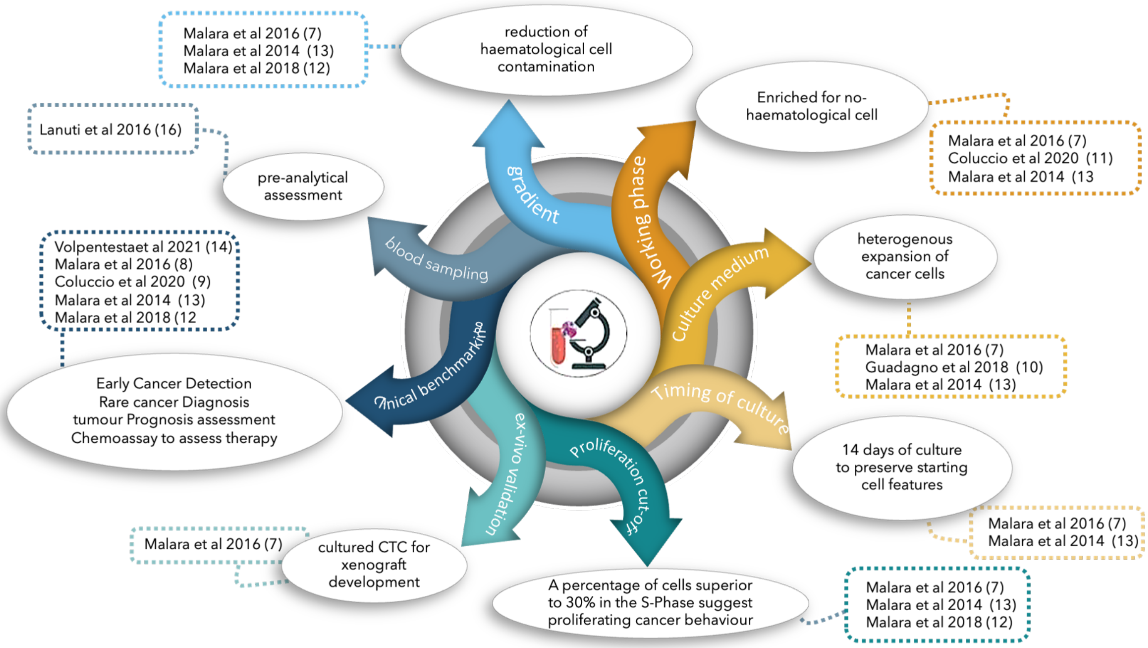

Figure S5: Published data to validate Charactex protocol. The graph summarizes the key points of the charactex protocol and the results of the part of the trial-tested in the work (s) published in this regard

- **Analysis.** The Charactex protocol generates cytologic preparations enriched for CTC in the function of their proliferative rate and virtue also of the impoverishment and not deprivation of the haematological cell fraction. These preparations offer the pathologists similar cellularity to the original cyto-histological diagnostic samples taken from the primary cancer lesions or their metastases. Differences lie in the numerical proportions among the cellular subgroups present in the sample and not in the absolute types of cells found in the preparations (which nevertheless have a heterogeneous cell composition). The identification of tumour cells is performed by the pathologist following traditional diagnostic criteria, independently from their context of observation. The cytomorphologic examination is followed by immunocytochemical staining for tissue or organ-specific markers whose expression is relevant for diagnosis [5]. Moreover, cell cultures on plates that show enrichment in tumour cells > 50% are a good source of cell pellets or cell blocks that can be used for mutational analysis by using rapid tests (e.g.: the Idylla test). As shown in figure S5, our laboratory validated the protocol through the publications shown in the figure based on the prevailing experimental data in the context of the protocol. In particular, the cytomorphological evaluation of the preparations was conducted to verify that the expanded cell population

was already identifiable by its morphological features before performing immunocytochemical tests. Moreover, the cytological evaluation also assessed the representativity of CTCs concerning the resident tumour population. Biomarker performance will be evaluated using sensitivity, specificity, positive and negative predictive values, likelihood area under the receiver operating characteristic curve (ROC). Figure S6 shows the ROC curves which indicate that among the criteria for the determination of the degree of cellular atypia on the cytological preparations, those adopted in the Charactex protocol are the ones with the greatest sensitivity and diagnostic specificity. Furthermore, this data was validated by a double-blind cytologic evaluation of the preparations carried out by three different dedicated Laboratories using the classification variables. The correlation between the centers was satisfactory in identifying the tumour diagnosis starting from the evaluation of the non-haematological component of the blood shortly expanded in vitro.

Validation of cytopathological variables

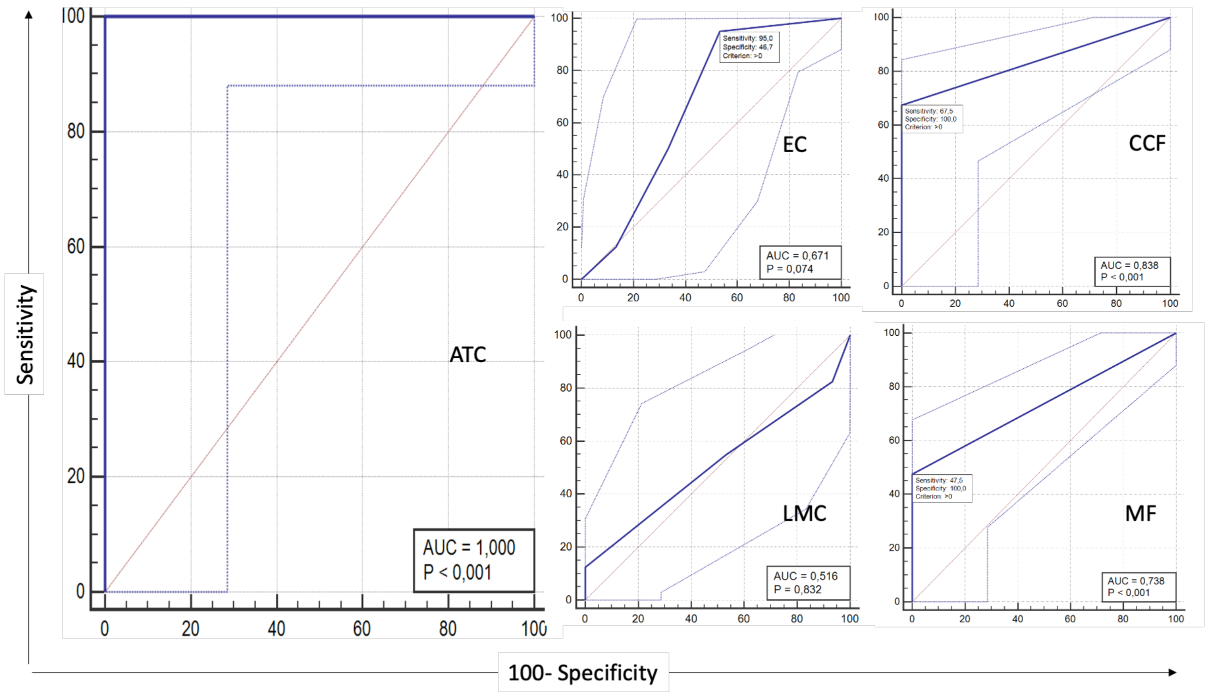

Figure S6: ROC curves of the cytopathological variables. Receiver operating characteristic curves, or ROC curves are graphical plots that illustrate the diagnostic ability of a binary classifier system as its discrimination threshold is varied. These latter are created by plotting the true positive rate (TPR) against the false positive rate (FPR) at various threshold settings for each cytopathological variable. Among the variables, the present plots consider the presence of cellular atypia as pathognomonic of a tumour cell population. The presence of cell atypia shows also in CTCs preparations a high specificity and sensitivity, similar to that of traditional cytological preparations.

Note ROC: Receiver operating characteristic; ATC: atypical cell, EC: endothelial cell; CCF cell cluster formation; LMC lymphomonocyte; MF mitotic figures. AUC; area under the curve

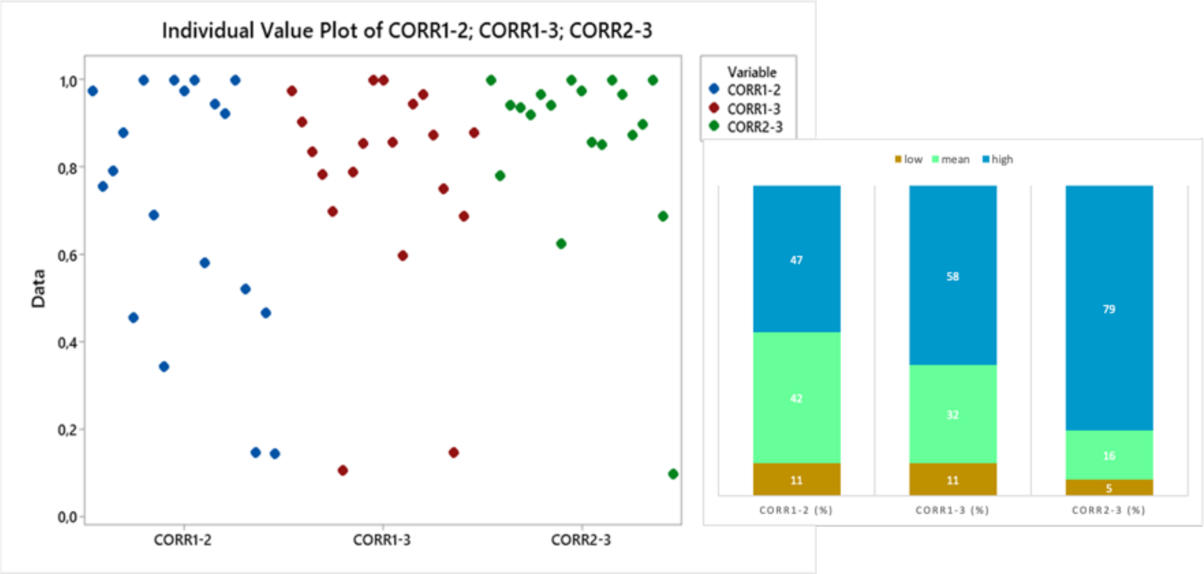

Figure S7: Multicenter validation. Three Cytology Centers independently evaluated the blood-derived cytological preparations based on the cytopathological variables with a different concordance degree of a cancer diagnosis. The evaluation performed on blood-derived cytological preparation by three independent pathologists in the three centers involved showed a Pearson correlation coefficient r>0.8
