# Supplementary material for: Multicancer screening test based on the detection of circulating non haematological proliferating atypical cells
Source: Mol Cancer. 2024 Feb 13;23:32. doi: 10.1186/s12943-024-01951-x (PMC10863189; doi:10.1186/s12943-024-01951-x)
Supplement: Supplementary file 5 — Supplementary Material 5 [file 12943_2024_1951_MOESM5_ESM.pdf]

Additional file 5 : Supplementary file.pdf. The predictive model .

Correspondence to: nataliamalara@unicz.it

This PDF file includes:

Figs. S11
Supplementary Text : Predictive model
Figs. S12 to S21
Table d

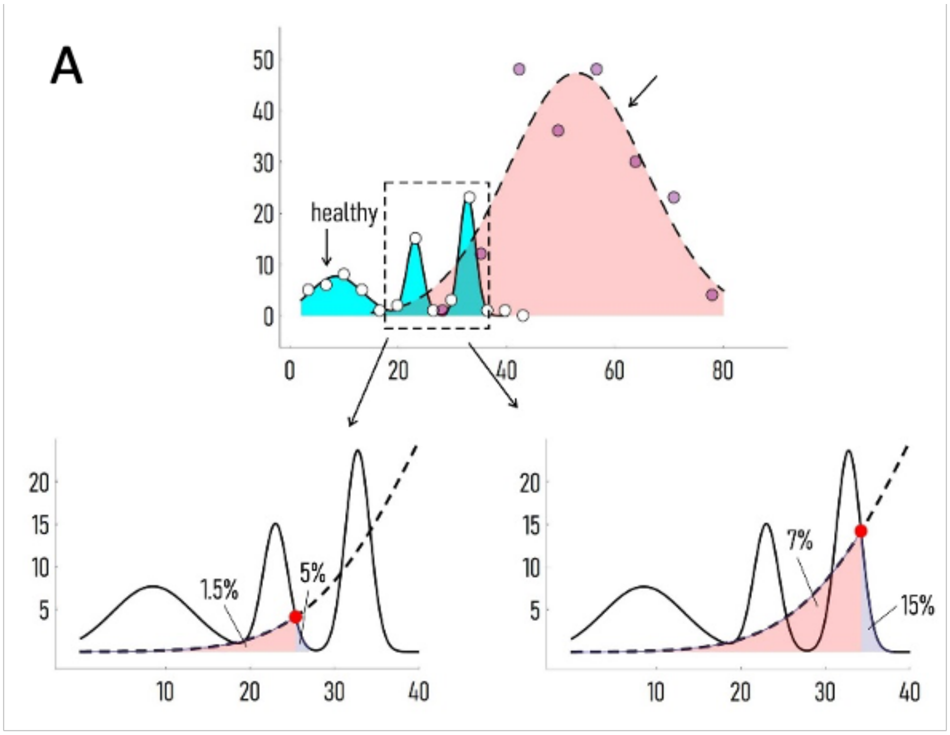

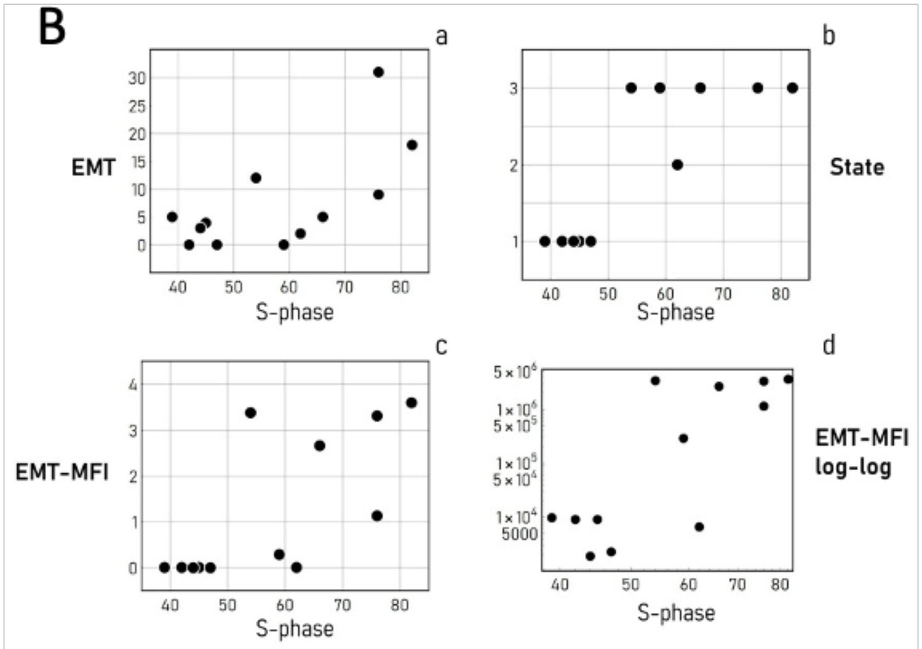

e

Pearson Correlation Test

	Statistic	P-value
**EMT**	0.6168	0.0187
**EMT-MFI**	0.5731	0.0321
**State**	0.8120	0.0004

Figure S11. Fitness of statistical criteria on operating characteristics curve based on the proliferating CTCs rate. A) Gaussian distributions were applied to fit the percentage of cultivated cells in S-phase or S-phase fraction data from CS and CP cohorts. The areas where the two profiles overlapped gave the total error of the analysis, indicating the false positive on the right-side respect to the intersection point of the two Gaussian distributions. The false negative is on the left-side of the same point. Consequently, considering as the limit of decision an S phase value ≤30%, the probability of false negative and false positive was respectively 1.5% and 5% (zoom in left-side). These values increased to 7% (false negative) and 15% (false positive) (figure, right-side) when the limit of decision (S-Phase) was pointed >30%. In B) comparative analysis of S-phase data with the entity of epithelial-mesenchymal transition expressed as a percentage of cells (EMT) p=0.01and as Median Fluorescent Intensity (MFI) p=0.03. The highest correlation was found between the S phase percentage and the cancer stage (p=0.0004).

Notes: Control subjects (CS) and Cancer patients (CP), Circulating tumour cells (CTCs), Epithelial-Mesenchymal transition (EMT), Median Fluorescent Intensity (MFI)

Predictive model

We present a protocol outlining a prospective validation study to evaluate the biomarkers' performance in predicting clinical outcomes of patients with cancer.
This prospective validation study assesses patients with cancer, in whom blood samples are prospectively collected. Recruited patients include a range of cancer patients from localized to advanced cancer patients, recruited from the community, outpatient clinics, departments and hospitals. Study samples consist of peripheral blood samples collected into cell living-preserving tubes on patient presentation or immediately on study enrolment. The Charactex protocol will be performed on cellular suspension extracted from collected blood samples using the gradient phase. The Charactex data will be analysed to assess the diagnostic performance of CTC's quantitative, qualitative and functional biomarkers in predicting cancer. This is a prospective observational study to validate cultured CTC biomarkers in a new cohort of patients with cancer. the study design adopted an "umbrella trials" scheme for investigating the utility of targeted interventions based on predictive circulating cell biomarkers. In Charactex the screening population is stratified into subgroups on predictive biomarkers (e.g., single histology cancer and healthy volunteers)

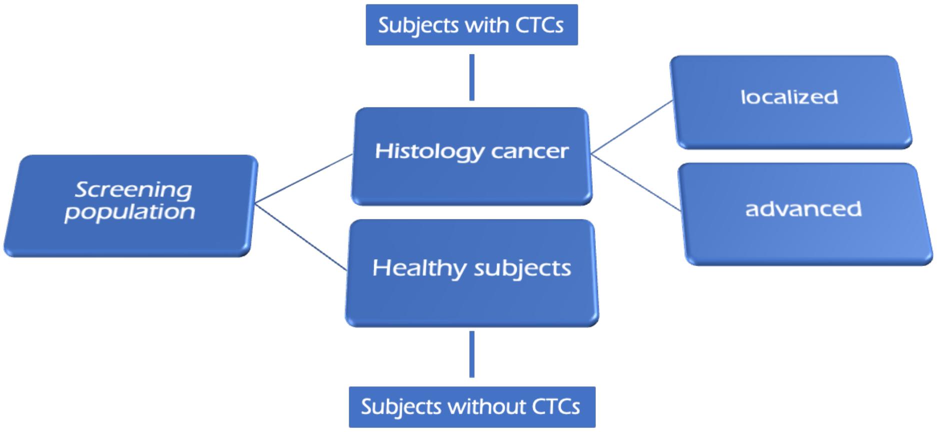

Figure S12: Prediction model study: Schematic representation of diagnostic modelling Charactex study "Umbrella design" based. The aim of the prediction in diagnosis by Charactex protocol is to estimate the probability that atypical cells are present within the blood sample of an individual at the moment of the prediction (T0).

Analytics data to construct a predictive model

The analytic data used to build the prediction model were qualitatively based on the cytopathological features of the cultured cells isolated from the blood samples of cancer and healthy subjects. The imaging scoring of epithelial and mesenchymal features of cancer cells transforms the qualitative data into a quantitative evaluation as reported in the text.

The cytopathological features of the cell culture obtained by applying the Charactex protocol are represented in figure S in which the different types of cells are stained to facilitate their identification. A further distinction is needed between localized and advanced cancer patients. In the cohort of cancer patients, the different extensions of the disease conditioned the density of CTCs present in the blood-derived cultures.

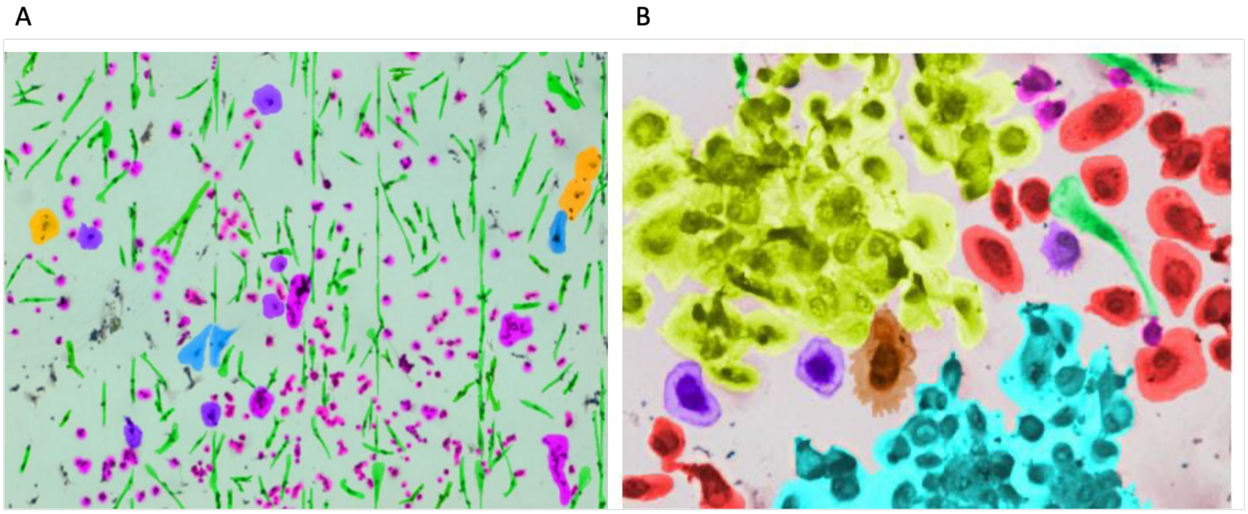

Figure S13: Qualitative variables of the model. Example image of cell qualitative variables corresponding to the cytopathological features of the cultured cells isolated by the Charactex protocol. Figure (A) shows cultured blood derived from the blood sample of a healthy subject. In (B) a case of breast cancer patient showing atypical cell enrichment.

Notes:
Endothelial cells
Histiocytes
Reactive stromal cell
Macrophages
Lymphomonocytes
Circualting Tumor Cells

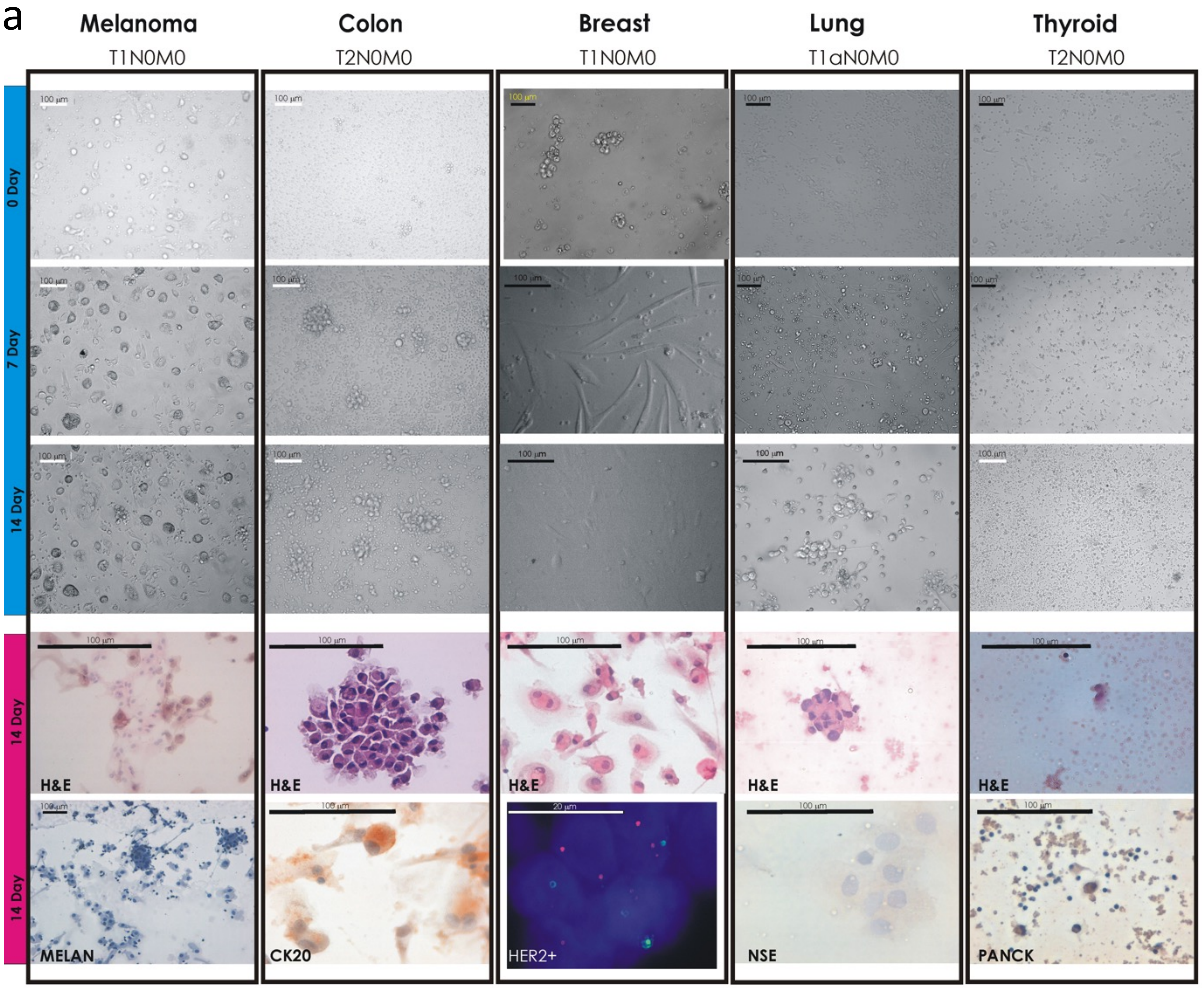

a
Melanoma
T1N0M0
Colon
T2N0M0
Breast
T1N0M0
Lung
T1aN0M0
Thyroid
T2N0M0
0 Day
7 Day
14 Day
14 Day
14 Day
H&E
H&E
H&E
H&E
H&E
MELAN
CK20
HER2+
NSE
PANCK

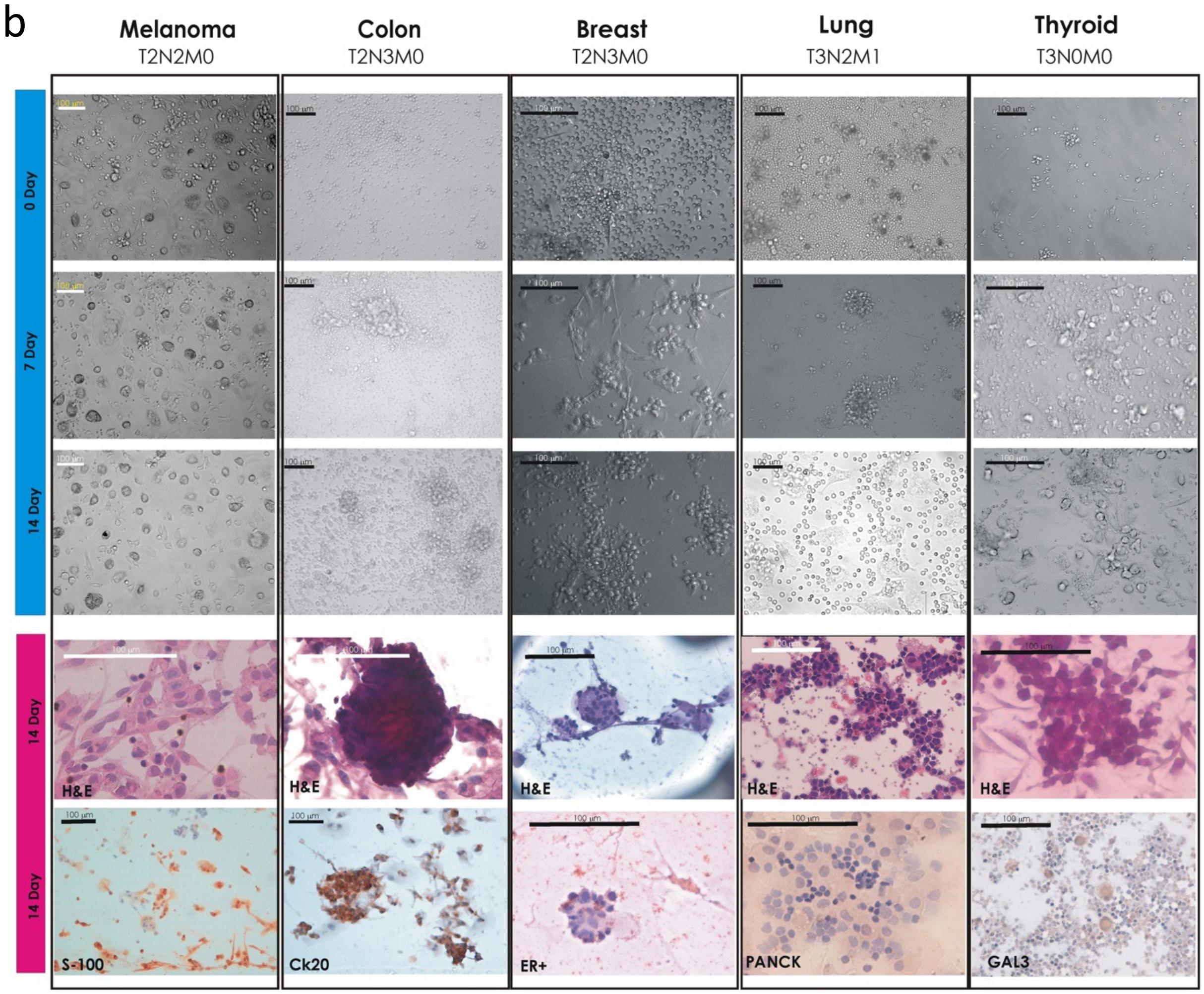

Figure S14: Cytopathological model from Charactex protocol in patients with localized and advanced disease: cellular features within the traditional protocol of cytopathological characterization of the blood-derived culture from CP in which the disease grown inside the primary localization (a) (localized) or outside the body part it started (b) (advanced cancer)

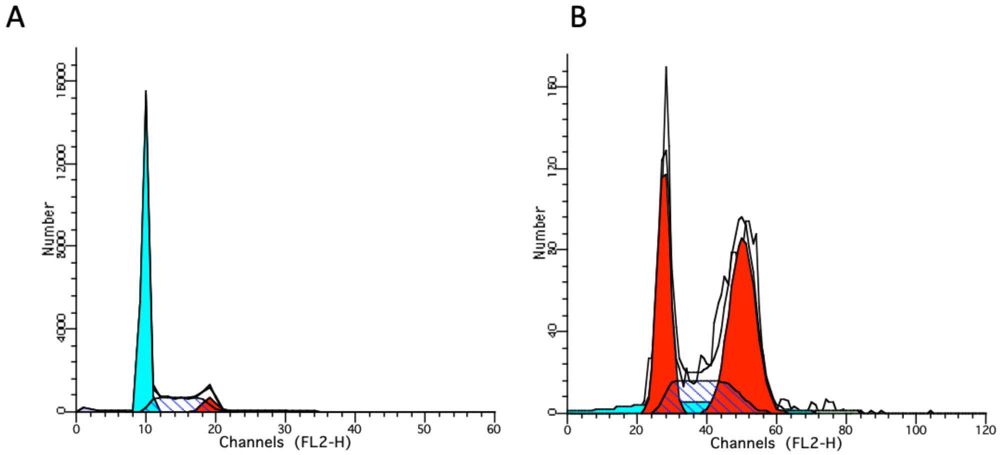

Figure S15: Quantitative data of the model. Examples of cell cycle phase distribution performed by labelling DNA with propidium iodide (PI) for fluorescence-based analysis of the cell cycle. This cytometric test generates a cell population histogram concerning PI fluorescent intensity. The amount of PI fluorescent intensity is correlated to the amount of DNA within each cell. Since the amount of DNA doubles (2n → 4n) between G1 and G2 phases the amount of fluorescent intensity of the cell population also doubles from 2000 to 4000 fluorescence units. In particular, the S-Phase corresponds to the phase in which the cell replicated its DNA before the successive cell division, for this reason, their fluorescent intensity value is intermediate between 2000 to 4000. In Figure (A) i.e. blood-derived cultured cells showing an S-phase of 28% in the CS cohort. In (B) i.e. 45% of the S phase percentage in the CP cohort.

Architecture of the network

The algorithm was based on artificial neural networks (ANN) with an input layer, an output layer, and 4 hidden layers, respectively composed of: (1) linear layer, (2) normalization layer, (3) hyperbolic tangent non-linear layer, (4) linear layer. The input layer was here represented by a set composed of one clinical variable (S-phase) plus two cytopathological parameters (Atc, CCF). The output layer was a Boolean layer with two possible 0\1 states. We used 4 neurons for the hidden layers, and 3 and 2 neurons for the input and output layers, respectively. The algorithms were implemented in Mathematica. The Mathematica NetInitialize function was used to instantiate the model. The algorithm was trained upon a dataset of 200 samples setting an epoch number of 1000 samples and a batch size of 256 samples. Since we used labelled datasets to train the algorithms to classify data or predict outcomes accurately, this is a supervised learning method.

Data Analysis Plan

Thus, the network was trained such that it visited each example 1000 times while processing up to 256 examples simultaneously. The values for the S-phase, Atc and CCF were concatenated to create a single feature set for each sample. In the tests, the error loss always converged to a value of steady state (~10^(-2)), indicating that the model achieved optimal performance for the considered values of model parameters. The algorithm was validated against a dataset of 60 samples. We measured the performance of the model using 3 different metrics: accuracy, sensitivity and specificity. Where accuracy is the proportion of correct predictions among the total number of examined cases; sensitivity is the proportion of positive results that are true positives; specificity is the proportion of negative results that are true negatives.

Benchmarking the AI model of cancer diagnosis

The values of sensitivity and specificity that we have found for the AI model are very high and close to one. To verify that this is not coincidental and that the performance of the model has a low or negligible sensitivity to the size and composition of the training and validation set, we performed additional test campaigns. For each campaign, we fixed the size of the training and validation sets, then we chose randomly the elements of the sets from an initial pool of 290

samples. Following this random choice, we trained the AI model using the training set and determined its sensitivity and specificity using the validation set. We selected the size of the training (validation) set as 120 (170), 140 (150), 160 (130), 180 (110), 200 (90), and 220 (70) data samples, such that their sum is constant and equal to 290, i.e. the size of the originating dataset. For each size, we performed 100 different tests. The scheme of the experiments is recapitulated in the **Table d.** The results of the tests are shown in **Figure S16**. In Figure S16, for each of the configurations considered for this study, we report the values of sensitivity and specificity resulting from 100 different simulations, in which the training and validation sets were generated randomly. We observe that for each configuration the points associated with the tests are scattered in the upper-right region of the diagram, exhibiting high values of sensitivity and specificity close to one. In **Figure S17** and **Figure S18,** we reported the density histogram, and smooth density histograms, relative to the described tests. These diagrams illustrate the distribution of data points with given values of sensitivity and specificity in the 0-1 interval. In **Figure S19**, the mean values of sensitivity and specificity resulting from different data-sample sizes are reported in the same plot. The error bars in the diagram are proportional to the standard deviations of sensitivity and specificity determined for each test campaign. The low dispersion of data points in **Figures S16-S18,** and of their means in **Figure S19**, illustrate that the performance of the AI model is only marginally influenced by the size and choice of the training and validation set. This is further evidenced by the whisker box plots of sensitivity and specificity derived for all the considered samples in **Figure S20** and **Figure S21.**
Statistical analysis of the datasets enabled to identify the median value of sensitivity as 0.926, 0.984, 0.982, 0.980, 0.955, 0.9642, for a size of the training (validation) set of 120 (170), 140 (150), 160 (130), 180 (110), 200 (90), 220 (70) elements, respectively. The same analysis performed in the same sample-size-range, enabled to identification of the following median values of specificity: 0.909, 0.890, 0.913, 0.923, 0.888, 0.875.
The 25% (75%) sensitivity and specificity values for the same distributions were instead determined as: 0.937 (0.994), 0.942 (1), 0.949 (1), 0.932 (1), 0.955 (1), 0.964 (1), regarding sensitivity, and 0.876 (0.943), 0.843 (0.933), 0.857 (0.955), 0.872 (0.962), 0.777 (0.944), 0.833 (0.937), about specificity.
The very low variation of sensitivity and specificity for all considered sets is a sign of the AI model's robustness.

Test campaign	Training set size	Validation set size	Number of tests
**1**	120	170	100
**2**	140	150	100
**3**	160	130	100
**4**	180	110	100
**5**	200	90	100
**6**	220	70	100

Table d. Scheme of the numerical tests performed to benchmark the AI cancer-diagnosis model.

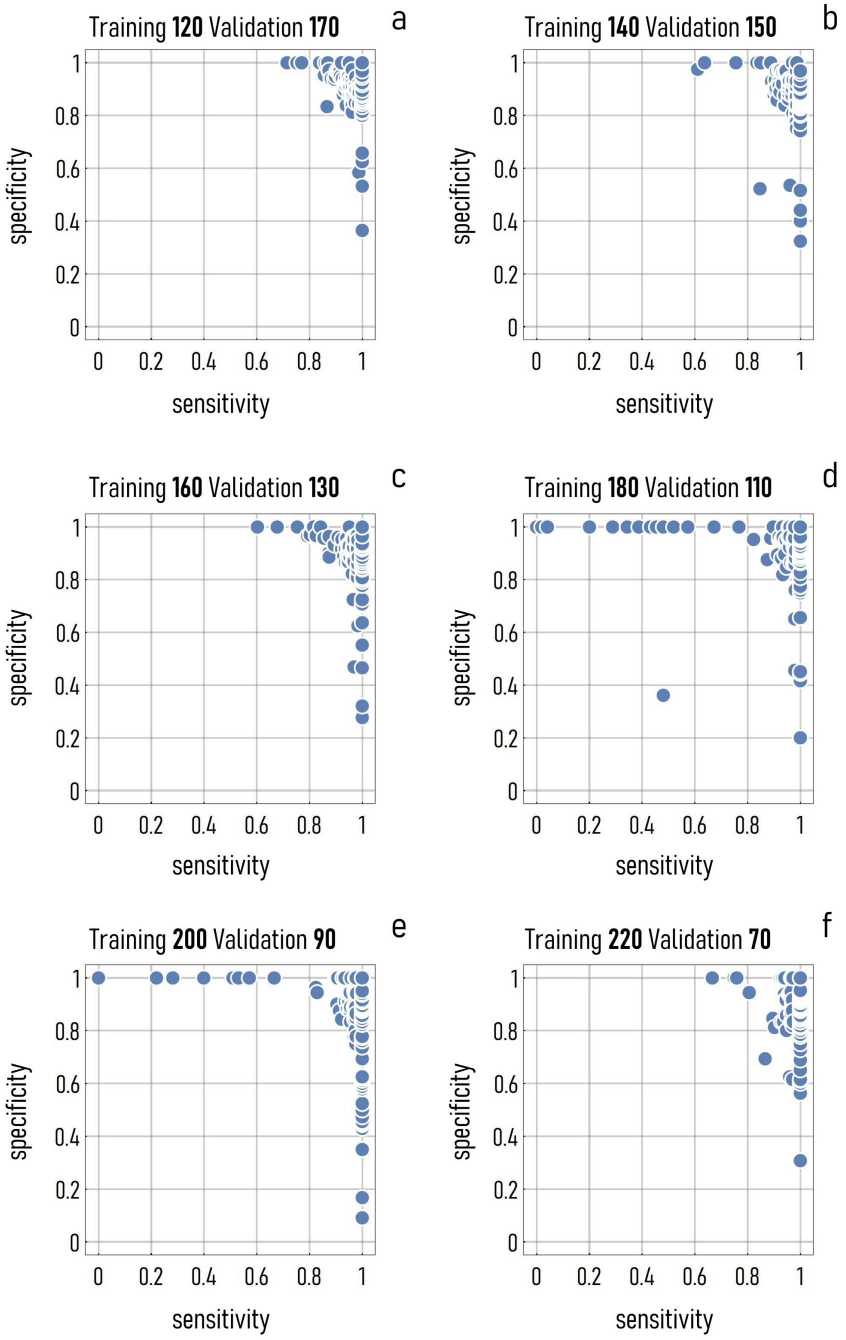

Figure S16. Evaluating AI model performance. Values of sensitivity and specificity determined for 100 different AI models, trained on randomized training sets, as a function of the size of the training and validation set.

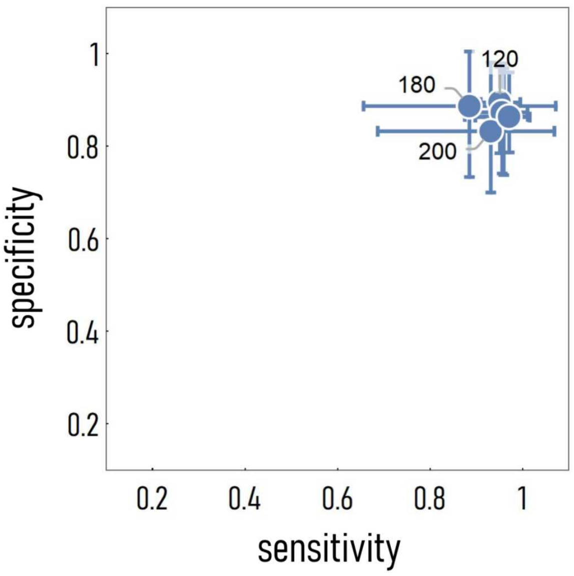

Figure S17. Mean values of sensitivity and specificity and AI performance.
Mean and standard deviation of the sensitivity and specificity determined over 100 different versions of an AI model of cancer diagnosis, for different sizes of the training and validation set. Each version differs from the other because of the random composition of the training and validation set.

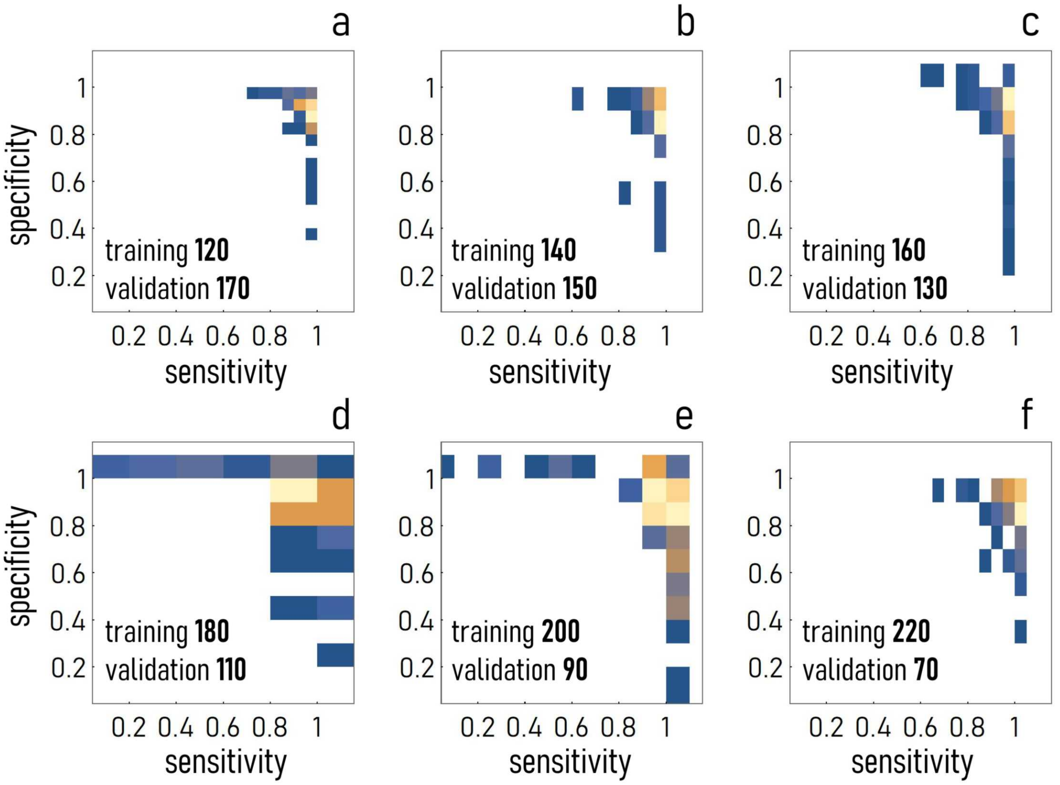

Figure S18. Sensitivity vs Specificity values (density plots).
Density plots of sensitivity vs specificity values of the AI model in stratifying healthy from non-healthy subjects as a function of the size of the training and validation sets. For each size, we performed 100 tests to assess performance of the AI model on a statistical basis.

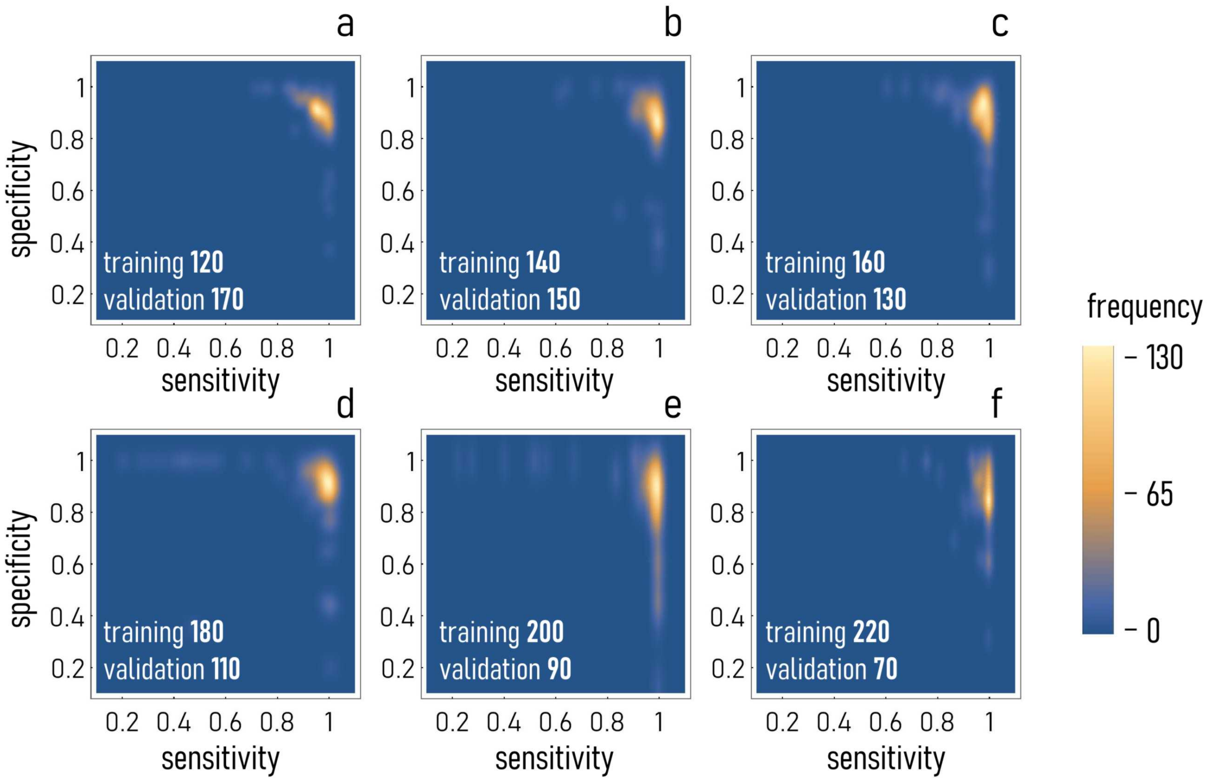

Figure S19. Sensitivity vs Specificity values (smooth density plots). Smooth-density plots of sensitivity vs specificity values of the AI model in stratifying healthy from non-healthy subjects

as a function of the size of the training and validation sets. For each size, we performed 100 tests to assess performance of the AI model on a statistical basis.

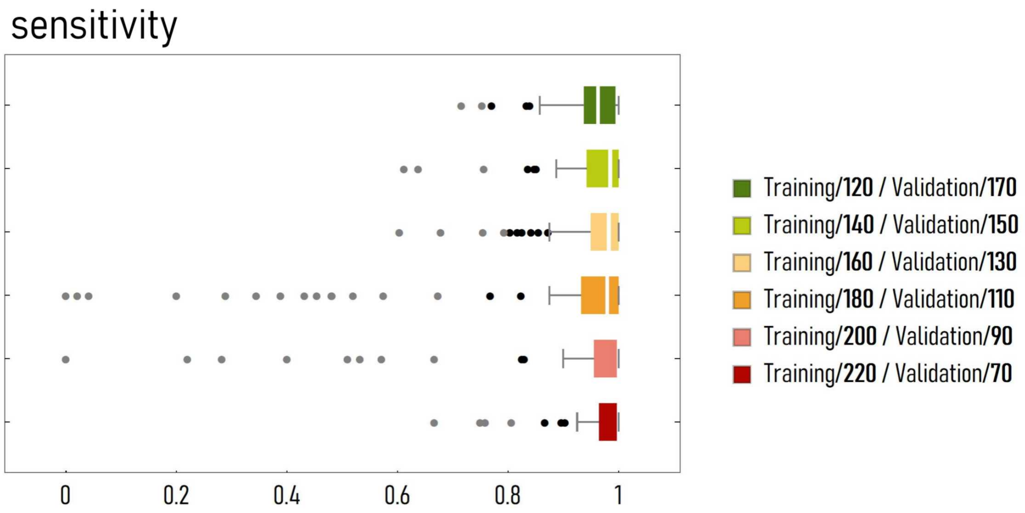

Figure S20. AI classification model on cancer and non-cancer patients. Whisker box plots of sensitivity of the AI classification model on cancer and non-cancer patients, as a function of the size of the training set and of the validation set.

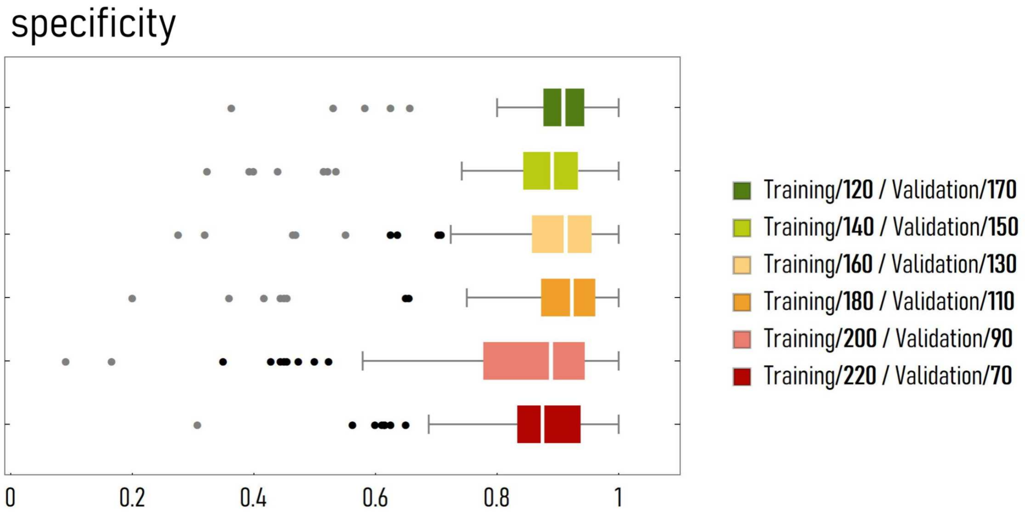

Figure S21. AI classification model on cancer and non-cancer patients. Whisker box plots of specificity of the AI classification model on cancer and non-cancer patients, as a function of the size of the training set and of the validation set.
