# Supplementary material for: Multicancer screening test based on the detection of circulating non haematological proliferating atypical cells
Source: Mol Cancer. 2024 Feb 13;23:32. doi: 10.1186/s12943-024-01951-x (PMC10863189; doi:10.1186/s12943-024-01951-x)
Supplement: Supplementary file 7 — Supplementary Material 7 [file 12943_2024_1951_MOESM7_ESM.pdf]

Additional file 7 : Supplementary file.pdf. Materials and Methods

Correspondence to: nataliamalara@unicz.it.

This PDF file includes: Materials and Methods

Patients Selection. The prospective project CHARActerization of Circulating Tumor Cells and EXpansion (CHARACTEX), was approved by the Regional Institutional Research Ethical Committee with the number 2013.34. The eligibility criteria of the study were reported online at http://www.bionem.unicz.it/web/. Patients with tumour diagnosis of carcinomas have been enrolled at the Health Sciences and Experimental and Clinical Medicine Departments at Magna Graecia University at Pugliese Hospital in Catanzaro, and San Giovanni Hospital Rome, (IT). Eligible patients were 18 to 90 years of age with diagnosed epithelial malignancies. Informed consensus on the general content of study methodology and objectives was collected from each patient entering the study, including the subjects belonging to the healthy cohort as a control group. Details of the Clinical Pathological findings of all patients and healthy subjects enrolled are listed in Table S1.
The inclusion criteria for our study are briefly reported as follows:
Cancer Patients Cohort (CHARACTEX Project Number: 2013.34)

- Caucasian race
- Patients with a confirmed diagnosis of non-haematological cancer
- Age between 18 and 85 years
- Contraceptive methods
- Written informed consent.

Healthy Subjects Cohort (CHARACTEX Project Number: 2013.34)

- Caucasian race
- Healthy and non-smoking adults
- Age between 18 and 85 years
- Absence of relevant diseases
- Contraceptive methods
- Written informed consent

Enrolment and diagnostic classification of the patients. Consecutive 205 patients were recruited (S1) of which 112 (54,4%) were females, and 93 (45,6%) were males with an age range of 21-90 years. In particular, 36 (17,6%) had a diagnosis of colorectal cancer (CRC), and 8 (3,9%) of glioblastoma (GBM). 3 patients (1,5%) were affected by head and neck carcinomas (HNC), 16 (8,3%) by hepatocellular carcinoma (HCC), and 15 (7%) by pancreatic ductal adenocarcinoma (PDAC). Moreover, 19 (9,3%) patients with lung cancer, of which 16 (87%) with non-small-cell-lung-cancer (NSCLC) and 3 (13%) with small cell lung cancer (SCLC). In addition, 47 (23%) with breast cancer (BC), 17 (8,4%) with cutaneous malignant melanoma (CMM), 2 (1%) with endometrial cancer (EC), 7 (3,4%) with ovarian cancer (OC), 14 (7,4%)

with prostate cancer (PC), 16 (7,8%) with thyroid cancer (TC), and 2 (1%) with cardiac myxoma (CM) and 1 (0,5%) with angiosarcoma (AS). Thirty-two (15%) of the recruited CRC patient tumours had genomic alterations suitable for monitoring (Table S4). Moreover, 72 control subjects were also enrolled, 22-88 years old of age, of which 30 (41, 7%) were females and 42 (58, 3%) were males. In this group 7 (2,8%) had a previous clinical history of carcinoma, 4 (5,6%) were affected by cardiovascular disease, 17, 23,6% were affected by chronic inflammation and 1 (1,4%) by hereditary/familial cancer disease. Samples 276 of peripheral blood were collected between December 18, 2013, and October 18, 2018, for the CHARACTEX project, 14 of the enrolled patients had no clinical evidence of cancer at the time of the blood collection. Of the 14 cases without current evidence of cancer, the application of the CHARACTEX protocol permitted to identification of 5 new cancer cases (1 thyroid undifferentiated cancer, 2 melanomas, 2 lung carcinomas: adenocarcinoma and NSCLC). Moreover 3 cases positive for CTCs developed cancer within 1 year (1 colon adenocarcinoma, 1 intraductal breast cancer, 1 ovarian cancer). These eight new cancer cases of cancer were positive for the presence of CTCs on cytological examination, immunocytochemical assay and mutational analysis and were characterized by an S phase>30%. All cases were confirmed by subsequent histopathological examination of the primary tumour. Moreover, 6 cases were identified which were positive for CTCs and characterized by a lower S phase (<30%) were identified. Four of these 6 cases became negative for CTCs within 1 year. The remaining 2 cases were repeated every 6 months for blood sampling and clinical check-ups. Their S-phase remains at low values (<30). In all cases with previous diagnoses of cancer, the cytological examination on CTCs confirmed the first diagnosis performed on tumour tissue. While being treated with chemo-radiotherapy, 32 patients repeated blood sampling. Tissue sections and CTC preparations from all of the 32 colon cancer patients were submitted to parallel mutational analysis for Kras mutations. Mutational analysis was performed on Formalin-fixed Cultured-Cell Pellets (FFCCPs) from CTC preparations. Blood sampling was repeated after chemotherapy on 32 colon cancer patients and, in 14 of these patients, the mutational status changed as reported in Table S4. Moreover, on 32-colon cancer patients, we performed comparative immunocytochemical staining for the expression of a diagnostic marker for CRC (CK20) between the cytological specimen and tissue biopsy. CK 20 expression was observed in both FFCCPs and tissue biopsy. Finally, as far as the 72 healthy volunteers, their healthy status was confirmed by the negativity for the absence or sporadic presence of atypical cells (0,5 cells/1000 cells) and a low proliferation profile (<30%).

Biological samples collection. Peripheral blood (5 ml into a tube containing EDTA) was collected from each patient. Samples were centrifuged to reduce the presence of cellular blood components. According to the heterogeneous size and relative cell density of cancer cells, the suspension enriched for cancer cells was isolated from the cell layer comprised between 1080÷1090 (g/m) density gradient values, as previously demonstrated by Malara et al [3-6].

Blood-derived cultures (BDCs). To develop the culture, cells of interest were isolated by working range as previously reported by Malara et al [3-6]. After washing in phosphate-buffered saline (PBS) the cells were recovered in a medium promoting in vitro expansion for 14 days (short-term cultivation). The medium composition was previously reported [3-6]. The CTC cultures were expanded for a limited time previously determined to be optimal at <_14 days [3-6]. This timing was chosen to permit quiescent CTCs pool to regain intrinsic proliferation ability.

In fact, in 11% of the cancer cases examined the first adhesion colonies were observed after the tenth day. A rich medium optimized for human cancer cells improves the plating efficiency of transformed cells when plated at low density. It was demonstrated that efficient cloning of CTCs, independently from the stage or type of tumour, supported cell viability in each phase of cancer development [3-6]. The threshold of cellular expandability was then set up analyzing the cell-cycle phase distribution. The results set the value of the S-phase (fraction of cells registered in the DNA Synthesis phase) at 30% as the cut-off value to discern between normal and transformed cells [3]. Moreover, in vivo, expanded CTCs were transfected to validate their tumorigenic ability [3]. Expanded CTCs were sub-collected based on the combination of in vitro behaviour and antigenic expression. The sphere-formation property, cell positive for the CD133 expression, and adherent cancer cells expressing CK-CXCL4 were investigated. These cellular subsets transferred in animal vectors showed different progression. Animal vectors treated with the injection of adherent cancer cells expressing CK-CXCL4 did not develop evident tumour lesions but showed general dissemination of CTCs into different tissues. On the contrary, the already known tumorigenic ability of CD133-CTCs concerning the other subsets was confirmed [3].

Cell cycle phases distribution analysis. Blood-derived cultures performed on plates were used. Cells (3-7) x105 have been washed with cold PBS and fixed with 70% ethanol at -20°C overnight. Pellets were suspended in 500 µl PBS containing 2 mg/ml RNAse (Sigma) and kept at 37°C for 60 minutes. Pellets have been stained as previously reported [5]. DNA content was analyzed by a FACScan (Becton and Dickinson San Jose, CA). Cell cycle phase's distribution was performed using the Becton Dickinson kit: CycleTEST plus DNA reagent Kit, data acquisition using FACS Canto II (Becton Dickinson) and the analysis was performed with ModFit LT software (http://modfit-lt.software.informer.com/4.0/) [3-6].

Cytopathology. The morphological assessment was performed with Haematoxylin and Eosin (H&E) and Diff-Quick staining. After 14 days of culture and regular collection of the conditioned medium, cells on slides were fixed. Slides were stained following a standard H&E and/or Diff-Quick staining and evaluated under a light microscope (Leica ICC50HD, Leica Microsystem, and Milan, Italy). Two pathologists experienced cancer disease, in each of the three specialized centres involved, interpreted without reference diagnosis assigning a cytopathological score.

Cytopathological scoring value applied on blood-derived cell cultures (BDCs). According to the original protocol, CTCs were isolated by the previously identified working-cell phase. The cells isolated from the working phase were seeded in a specific culture medium and expanded for at least 14 days. Parallel cultures were performed in chamber-slide and Petri dish for each patient, and were evaluated by three distinct specialized centres. The considered pathological variables were: the rate of lympho-monocytes (Vc1) and endothelial cells (Vc2), the presence of cytomorphological abnormalities (atypical cells) (Vc3), mitotic figures (Vc4), formation of homotypic (cancer) cell clusters (Vc5) formation of heterotypic (admixed neoplastic and inflammatory cells, histiocytic cells included) cell clusters (Vc6) presence of mononucleated histiocytes (Vc7) presence of multinucleated histiocytic cells (Vc8). Individual scores for each variable were estimated according to the following scoring system (i.e. 1-5 atypical cells/100 cells: score 1; 5-10 atypical cells/100 cells: score 2; >10 atypical cells/100 cells: score 3).

Immunocytochemical analysis
After 14 days cultured cells on slides were fixed. Immunocytochemistry was performed following the protocol previously described [5]. Briefly, slides were submerged in either sodium citrate buffer or Tris–EDTA buffer for heat-induced epitope retrieval at 97 ◦C for 20 min. Staining with primary antibody detailed in Table S3, was used.

Immunofluorescence. For cellular immunofluorescent staining, cells on slides were fixed. The slides were washed three times with PBS and then blocked with PBS containing 0.2% Triton X-100 and 10% bovine serum albumin for 1 h. Subsequently, the cells were then incubated with primary antibody (1: 1000) for 16 h at 4°C and washed three times with PBS. Cells were then incubated with Alexa Fluor 633 - or Alexa 488-conjugated secondary antibody for 3 h at 22°C and examined using a laser confocal microscope (Nikon TI-E). The TI-E microscope software was used to morphometrically analyse the fluorescence intensity.

Cytometry. Cultured cells were analyzed with a panel of antibodies designed to identify different populations of naïve haematological and non-haematological cells (the list of antibodies used in panel and control tubes is described in Table S3). The panel included markers non-specific to cancer cells, which, however, provided relevant information in the multipanel context. Cells were fixed and permeabilized using the BD IntraSure kit.
All antibodies were titrated to obtain optimal dilution for the present experimental setting. Instrument performances and data reproducibility were checked using a Cytometer Setup and Tracking Module (BD) and further validated through the acquisition of Rainbow Beads (BD). Compensations were calculated using CompBeads (BD) and single stained fluorescent cells for DNA dyes (Syto16 and 7ADD). Flow cytometry was performed with a FACS ARIA III (Becton Dickinson)

Statistical Analysis
All data are reported as means ± standard deviation except for cytometric data which are reported as means ± standard error. A three-way analysis of variance (ANOVA) was used to explore the relationship among the expression of cell markers, clinical parameters and tumour characteristics. The analytical comparison between patients and the control group was performed using Mann-Whitney and Kolmogorov-Smirnov tests with a valid statistical significance of $p < 0.05$. Sub-groups were compared using the T-test (for continuous variables) and Chi-square test or Fisher test (for categorical variables). Follow-up of the patients was defined as the number of months from diagnosis until the first occurrence of loco-regional or distant relapse or death if any of these occurred. Survival analysis has been conducted using the Kaplan-Meier method, log-rank test. All statistical analyses were performed using MedCalc for Windows, version 18 (MedCalc Software, MariaKerke, Belgium). For hierarchical clustering, pairwise distances among clinical parameters such as age, sex and status within the control group and stage, grade, sex and age, within the patient group, were calculated using correlation coefficient and Euclidean distance. Dendrograms were generated using Ward's method. Cytometry data were analyzed using a multivariate mathematical approach to identify several cellular patterns across the different patients. For this purpose, 5000 cells representing grade I, grade II, grade III, and grade IV patients and healthy individuals (summing up to 25000 cells) were extracted from FACS data. For each cell, the scattering intensities of 10 fluorophores were recorded. A principal component

analysis (PCA) was applied to the whole fluorimetric dataset, and a K-means clustering analysis (KCA) was used to disclose a classification pattern among the cells. In the figures, heatmaps show the different patterns as a function of the average fluorophore intensities. Although a large number of patterns affected the analysis generating overlapping classes in the heatmaps, seven distinct patterns were identified. The first three principal components (PCs) are employed to show 3D scatter plots of all the cells, highlighting both the pattern distribution across the patients and the normalized fluorophore intensities.

The predictive method adopted for the algorithm used is described in Additional file 5
